# Atomic Force Microscopy‐Based Nanomechanical Signatures for Staging Classification and Drug Response in Pulmonary Fibrosis

**DOI:** 10.1002/smll.202504526

**Published:** 2025-09-27

**Authors:** Andreas Stylianou, Katerina Polemidiotou, Vassilis Alimisis, Fotios Mpekris, Chrysovalantis Voutouri, Meropi Mari, George Filippidis, Andreas Zachariades, Stylianos‐Vasileios Kontomaris, Paul P Sotiriadis, Vasiliki Gkretsi, Triantafyllos Stylianopoulos

**Affiliations:** ^1^ Cancer Mechanobiology and Applied Biophysics Group Basic and Translational Cancer Research Center (BTCRC) School of Sciences European University Cyprus Nicosia 1516 Cyprus; ^2^ EUC Research Center Nicosia 1516 Cyprus; ^3^ Department of Electrical and Computer Engineering National Technical University of Athens Athens 15780 Greece; ^4^ Archimedes/Athena RC Marousi 15125 Greece; ^5^ Cancer Biophysics Laboratory Department of Mechanical and Manufacturing Engineering University of Cyprus Nicosia 1678 Cyprus; ^6^ Cancer Genetics Therapeutics & Ultrastructural Pathology Department The Cyprus Institute of Neurology and Genetics Nicosia 1683 Cyprus; ^7^ Institute of Electronic Structure and Laser Foundation for Research and Technology‐Hellas Heraklion Crete 71110 Greece; ^8^ Nicosia Lung Center Strovolos Nicosia 2012 Cyprus; ^9^ Biomedical Sciences Program Department of Life Sciences School of Sciences European University Cyprus Nicosia 1516 Cyprus; ^10^ Cancer Metastasis and Adhesion Group Basic and Translational Cancer Research Center (BTCRC) European University Cyprus Nicosia 1516 Cyprus

**Keywords:** biomarkers, bleomycin, mechanobiology, pirfenidone, support vector machines

## Abstract

Current therapies for pulmonary fibrosis (PF) show high variability in effectiveness, while the lack of specific biomarkers hinder early diagnosis, and treatment monitoring. With few approved drugs and inconsistent treatment responses, developing personalized therapies is essential. Since PF progression involves changes in tissue structure and mechanics, mechanobiology plays a central role. It is hypothesized that Atomic Force Microscopy (AFM) can identify unique nanomechanical fingerprints (NMFs) to characterize fibrosis stages and monitor treatment response. AFM is first used to assess NMFs in human biopsy samples, followed by evaluation in bleomycin‐induced murine PF models. NMFs are also studied in mice treated with pirfenidone, a drug known to reduce collagen I. AFM data are supported by histopathological staining, polarized and second harmonic generation (SHG) microscopy, and real‐time PCR analysis of collagen I expression. AFM measurements detected distinct NMFs in human samples and tracked alterations during PF progression in mice. Changes in NMFs correlated with collagen I content, histology, and SHG microscopy. In silico analysis supported NMFs’ potential as diagnostic biomarkers. Furthermore, AFM‐based NMFs assessed pirfenidone treatment outcomes. These findings provide the first evidence that AFM‐based NMFs can serve as biomarkers for PF staging and treatment monitoring, offering a powerful diagnostic tool to complement standard biopsies.

## Graphical abstract

Atomic Force Microscopy (AFM) reveals nanomechanical fingerprints (NMFs) that track the progression of pulmonary fibrosis (PF) and response to therapy. These NMFs correlate with collagen I content and gene expression, offering a novel biomarker for disease staging and treatment monitoring. This study highlights AFM's potential to improve diagnosis and personalize care for PF.

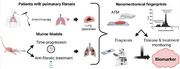

## 1 Introduction

Pulmonary fibrosis (PF) is a progressive and often fatal lung disease characterized by deposition of excessive amounts of extracellular matrix (ECM) in the lung leading to lung scarring, which ultimately impairs normal respiratory function.^[^ ^1^, ^2^ ^]^ The efficacy of current standard therapies for PF is highly variable, owing to both intra‐ and inter‐patient differences while the lack of reliable biomarkers capable of accurately characterizing the PF stage prevents effective diagnosis, prognosis and treatment monitoring.^[^ ^3^, ^4^ ^]^ This variability in effectiveness is greatly dependent on correct staging and presents a significant challenge in PF treatment, as only a subset of patients responds well to the currently approved therapies.^[^ ^5^, ^6^ ^]^ Thus, there is an urgent need for the development of novel biomarkers able to accurately characterize the stage of the disease, and facilitate disease monitoring while also predicting response to treatment in each individual patient.^[^ ^7^, ^8^, ^9^, ^10^ ^]^

Given that fibrosis is characterized by excessive deposition of ECM components, mainly collagen type I, that leads to significant tissue stiffening, disrupted alveolar architecture, thickened interstitial spaces, and the acquisition of an altered mechano‐cellular phenotype, mechanobiologybecomes more and more relevant.^[^ ^11^, ^12^, ^13^ ^]^ Mechanobiology examines the interplay between mechanical properties and biological processes, thus offering a unique tool for assessing disease states that include such alternations in mechano‐cellular phenotype.

Traditionally, regular histopathological and radiological data along with gene expression analyses are employed to assess the state of fibrotic tissues, like PF (including idiopathic pulmonary fibrosis‐IPF),^[^ ^14^, ^15^ ^]^ yet the potential of nanomechanical properties as biomarkers is usually overlooked.^[^ ^16^ ^]^

Interestingly, recent advances in atomic force microscopy (AFM), a fundamental tool in mechanobiology, have enabled the assessment of mechanical properties at the cellular level. In fact, more recently, the application of AFM for the mechanical characterization of tissue biopsies has shown promise for diagnostic purposes.^[^ ^17^, ^18^, ^19^, ^20^ ^]^ However, most of these research efforts are mainly focusing on cancer, with limited studies in PF.^[^ ^21^, ^22^ ^]^

Utilizing knowledge acquired from previous studies, where we successfully used AFM to optimize the dose of the dual endothelin receptor antagonist, bosentan, as a tumor microenvironment (TME) normalization agent,^[^ ^23^ ^]^ we hypothesized that AFM could identify unique nanomechanical fingerprints (NMFs) that have the potential to serve as novel biomarkers for the PF characterization, and diagnosis, as well as for treatment monitoring.

In the present study, we employed a combination of cutting‐edge experimental approaches to test this hypothesis. First, we validated the ability of AFM to measure nanomechanical properties in human lung biopsies from PF patients. We then extended our investigations to a bleomycin‐induced murine model of PF. A comprehensive set of techniques, including AFM, histology, non‐linear microscopy, and gene expression analysis, was used to assess NMFs at various stages of disease progression. Additionally, *in silico* analysis (using Support Vector Machine, SVM) was performed to classify the data obtained from AFM and optical microscopy. Finally, we evaluated the efficacy of NMFs as biomarkers for monitoring treatment responses by treating fibrotic mice with pirfenidone, one of the few approved drugs for PF treatment.^[^ ^5^ ^]^

## 2 Experimental Section

### 2.1 Human Biopsies

Human lung specimens from biopsies taken from 5 PF patients were obtained from Nicosia Lung Center (Nicosia, Cyprus) through routine bronchoscopy performed for diagnostic purposes. All procedures and experimental protocols were approved by the Cyprus National Bioethics Committee (licenses EEBK/EΠ/2022/04). Written informed consent was obtained from all individual participants included in the study. Patient lung tissue specimens were collected in accordance with the ethical approval EEBK/EΠ/2022/04, with inclusion criteria allowing adult individuals newly diagnosed with PF who consented to provide surplus tissue from diagnostic procedures; exclusion criteria comprised minors, individuals with disabilities, or those unable to provide informed consent. Due to the limited availability of specimens during the collection period, no further exclusion criteria were applied. Six human lung specimens were analyzed, which reflects the limited availability of biopsy‐confirmed PF cases in Cyprus. Additionally, several eligible patients were not willing to provide informed consent or donate tissue specimens, which further limited our ability to increase the sample size. The samples from these cases, however, exhibit consistent mechanical differences. Biopsies were immediately placed in ice‐cold phosphate buffered saline (PBS) supplemented with a protease inhibitor cocktail (Complete Mini, Roce Dianostics GmbH, 1 tablet per 10 mL)^[^ ^19^, ^20^ ^]^ and kept at 4 °C to minimize tissue degradation. AFM analysis was performed no later than three days after biopsy.

### 2.2 Animal PF Model and Experimental Protocols

Mice were purchased from the Cyprus Institute of Neurology and Genetics (CING), and all *in vivo* experiments were conducted in accordance with the animal welfare regulations and guidelines of the Republic of Cyprus and the European Union (European Directive 2010/63/EE and Cyprus Legislation for the protection and welfare of animals, Laws 1994–2013). All animal experimental protocols were approved by the Cyprus Veterinary Services, the Cyprus national authority for monitoring animal research (licenses No CY/EXP/PR.L03/2022). For this study the bleomycin‐induced PF model was used as described previously.^[^ ^24^, ^25^ ^]^

#### 2.2.1 Drugs and Reagents

For *in vivo* studies, pirfenidone (Esbriet, Roche Pharmaceuticals, Switzerland), an approved anti‐fibrotic and anti‐inflammatory drug for IPF, was dissolved in sterile water and heated to 60 °C for 30 min to ensure complete solubilization.^[^ ^26^ ^]^ Bleomycin (Bleomycin sulfate, derived from Streptomyces verticillus, #B5507, Sigma,) was prepared by dissolving 15 units (U) (from a 9.4 mg solid powder) in PBS to a final concentration of 3 U mL^−1^.

#### 2.2.2 Development of Mouse PF Model

Bleomycin was administrated intratracheally, as previously described.^[^ ^27^, ^28^ ^]^ Briefly, 6–8 week old C57BL/6 female mice were anesthetized with intramuscular ketamine (80 mg kg^−1^) and xylazine (15 mg kg^−1^) and bleomycin was injected intratracheally (2U/kg).^[^ ^24^ ^]^ Mice were then sacrificed at different PF stage via CO_2_ inhalation and lungs were excised for analysis. Specifically, mice were euthanized during the: 1) acute injury and inflammatory stage (1‐7 days post bleomycin administration), 2) transition stage from inflammation to active fibrosis (7‐14 days post of bleomycin) and 3) chronic fibrosis stage when intra‐alveolar and septal fibrosis becomes morphologically evident (after 3–4 weeks post of bleomycin).

#### 2.2.3 Treatment with Pirfenidone

Following the protocol applied in the clinic, where PF patients receive pirfenidone orally, mice were given pirfenidone by oral administration of a single daily dose of 500 mg kg^−1^,^[^ ^26^ ^]^ starting at specific time points that correspond to different stages of the bleomycin‐induced PF model. Mice were sacrificed 7 days later and lungs were excised for analysis. The experiment included four treatment groups that selected to correspond to different stages of fibrosis progression so that a comparison could be made between healthy, treated and untreated fibrotic controls. Specifically, the groups were: i) Control‐Healthy lungs (treated with saline), ii) Control‐Fibrotic lungs (treated with saline), iii) Pirfenidone “*preventive*” treated‐fibrotic lungs (mice treated with pirfenidone during inflammation stage and 7 days post‐bleomycin administration) and iv) Pirfenidone “*therapeutic*” treated during active fibrosis stage‐fibrotic lungs (mice treated with pirfenidone between 7–14 days post bleomycin administration). More specifically, 21 control mice (5 at D3, 10 at D14, 6 at D21), 23 mice treated with bleomycin (5 at D3, 11 at D14, 7 at D21), and 6 mice per treatment group with pirfenidone were included.

**Figure 1:**
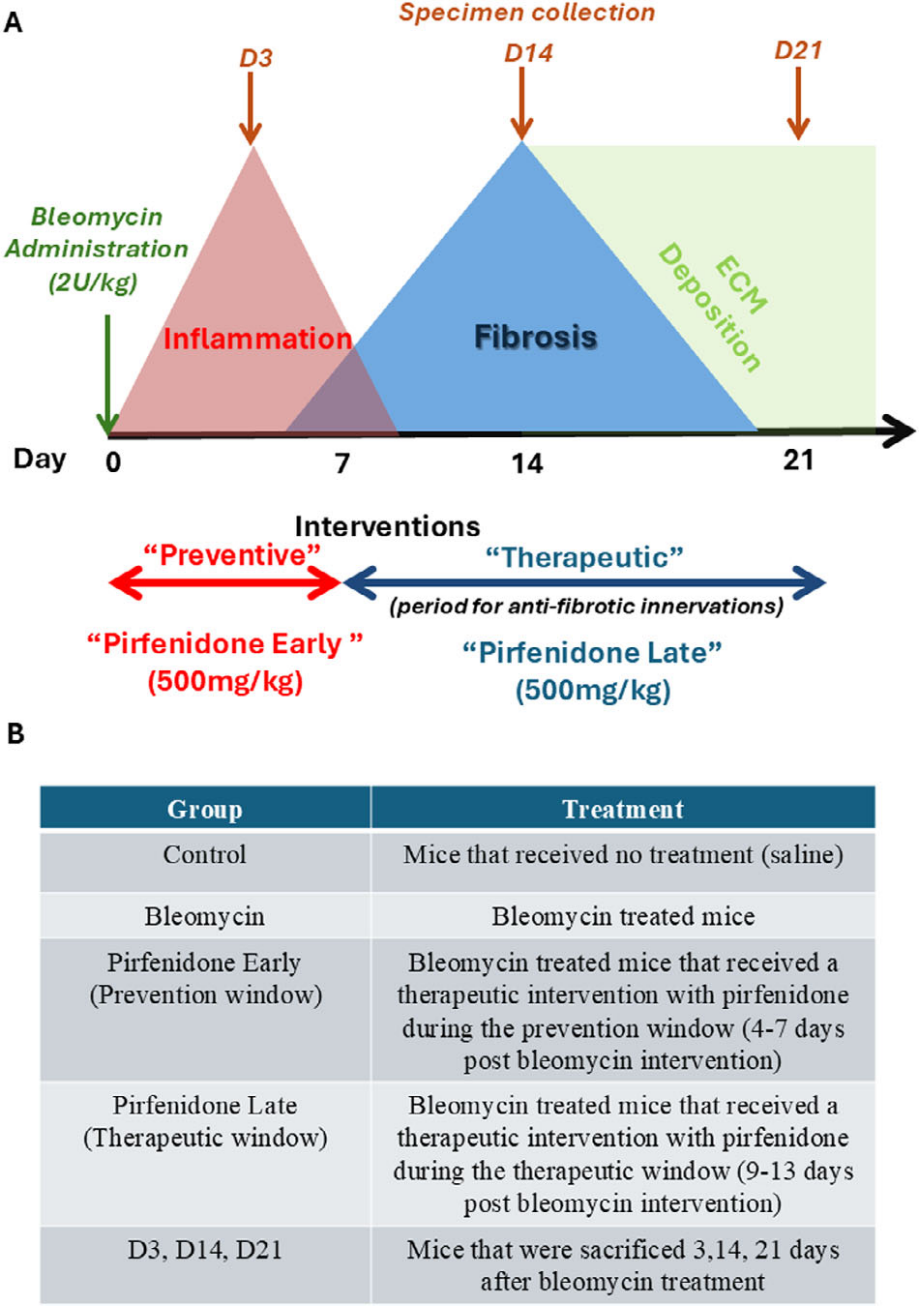
Experimental Protocol. A) Schematic representation of the experimental protocol. The different time points represent: 1) acute injury and inflammatory phase (1‐7 days post bleomycin administration), 2) transition phase from inflammation to active fibrosis (7‐14 days post of bleomycin) and 3) chronic fibrosis stage when intra‐alveolar and septal fibrosis becomes morphologically evident (3‐4 weeks post of bleomycin). B) Table summarizes the protocol's key parameters, relevant terms, and associated nomenclature.

An illustration of the experimental protocol for the in vivo experiments is shown in **Figure** **1** below.

### 2.3 AFM Measurements of Lung Nanomechanical Properties

AFM measurements were performed based on previously published protocols.^[^ ^17^ ^]^ More specifically, after human or murine biopsies were harvested, they were immediately transferred into ice‐cold PBS supplemented with a protease inhibitor cocktail as described above.^[^ ^19^, ^20^ ^]^ Then each specimen was immobilized on a 35 mm plastic cell culture petri dish with a thin layer of two‐component fast drying epoxy glue. The petri dish was filled with PBS supplemented with a protease inhibitor cocktail and stored at 4 °C to avoid tissue degradation.^[^ ^19^, ^20^ ^]^ AFM measurements were taken with a commercial AFM system (Molecular Imaging‐Agilent PicoPlus AFM), 1–72 h post tumor removal to prevent any alterations in stiffness profiles.^[^ ^19^, ^20^ ^]^ The measurements were taken with silicon nitride cantilevers (MLCT‐Bio, cantilever D, Bruker Company with the half‐open angle of the pyramidal face of θ ∼ 20°, tip radius: 20 nm, frequency in air: 15 kHz). The maximum applied loading force was set to 1.8 nN, the exact spring constant *k* of the cantilever was determined before each experiment using the thermal tune method and the deflection sensitivity was determined in fluid using petri dishes as an infinitely stiff reference material.^[^ ^29^ ^]^ To ensure consistent AFM calibration, measurements were cross‐checked using collagen‐coated hydrogel standards with defined Young's modulus values (0.5 kPa and 1.0 kPa, Petrisoft 35 mm Dish Collagen, Cell Guidance Systems), as described previously.^[^ ^30^ ^]^ These hydrogels were used as quantitative reference standards to ensure the accuracy of the measured modulus values. To assess potential AFM tip degradation and contamination, a test grating (TGT1, NT‐MDT) was scanned before and after the measurements to visualize the tip shape and confirm consistent performance throughout the experiments, according to the procedure reported in.^[^ ^31^ ^]^ All AFM measurements were performed under liquid conditions directly on 35 mm plastic Petri dishes to preserve tissue hydration and minimize sample degradation during data acquisition. AFM measurements were taken by recording 10–15 different 20 × 20 µm^2^ force maps (16 × 16 point grids) per specimen, which corresponds to 256 force‐displacement curves per map (up to 3840 force‐displacement curves per specimen) with pixel size of 1.25µm. Young's modulus was calculated using the Sneddon's equations for perfect conical indenters which is an extension of the classic Hertz mechanics.^[^ ^32^, ^33^ ^]^ The Poisson ratio was set to 0.5. and the collected force maps were analyzed by AtomicJ.^[^ ^34^ ^]^

### 2.4 Collagen Content Measurement (Polarized Microscopy and Collagen I and III)

Collagen content in murine lung specimens was assessed via picrosirius red staining. Briefly, fixed lung samples were dehydrated through a series of graded ethanol washes and embedded in paraffin. Transverse 7µm‐thick paraffin sections were produced using a microtome (Accu‐Cut SRM 200, SAKURA), flattened out into water and allowed to dry overnight at 37 °C. Sections were then deparaffinized, washed in ddH_2_O and stained with picrosirius red stain (ab150681, Abcam) for 1h at room temperature. Next, tissue sections were rinsed twice with acetic acid, followed by absolute ethanol and finally, mounted with DPX mountant for histology (Sigma). Collagen fibers were stained in red while the remaining tissue was pale yellow. Images of stained tumor sections from the tumor interior and periphery were acquired at 10x (Olympus UPlanFL N 10X/0.30na) and 20x (Olympus UPlanFL N 20X/0.50na) magnification using an Olympus BX53 microscope, without polarizers and with linear polarizers (U‐POT polarizer, U‐ANT analyzer, U‐KPA intermediate attachment, Olympus Corp.). To enable quantification, images of the same staining were taken at identical settings. All images were digitally captured using cellSens acquisition platform and analyzed using custom and built‐in algorithms in MATLAB.^[^ ^31^, ^35^ ^]^

### 2.5 Non‐Linear Microscopy

To confirm collagen distribution in the samples, second‐harmonic generation (SHG) signals in forward mode were detected, as described previously.^[^ ^36^ ^]^ Briefly a femtosecond laser beam (Coherent – Axon, 1064nm, 150fs, 80MHz) was guided to a modified upright microscope (Nikon Eclipse). Fast raster scanning in the selected xy plane of the sample was achieved using a set of galvanometric mirrors (Cambridge Technology). The focal plane was adjusted with a motorized translation stage (Standa Ltd.). A high numerical aperture objective lens (Carl Zeiss, 20Χ, NA 0.8, dry) was used to achieve diffraction limited focusing. Sample scanning and data acquisition were controlled through a LabVIEW interface adapted to the experiment requirements. For the detection of SHG signals in the forward path a condenser lens (Carl Zeiss, 40Χ, NA 0.8, dry), a proper bandpass interference filter (Thorlabs 532), and a photomultiplier tube (PMT Hamamatsu H9305‐04) were utilized. The laser power at the sample plane was kept below 1 nJ per pulse for all measurements. Typical time duration for obtaining a single 2D 500×500 pixels (218×218 µm^2^) SHG image was 1 second. To improve the signal to noise ratio (SNR), each final image was obtained by averaging 20 scans.

### 2.6 *In Silico* Studies: Classification Techniques

To substantiate the application of NMFs as potential biomarkers, we conducted a series of in silico experiments. For the experiments we used three types of data sets: 1) Optical microscopy images from picrosirius red stained tissues. 2) AFM‐based measurements (in terms of Young's modulus values) from lung tissues and 3) Optical microscopy data + AFM‐based measurements. For these data sets we had two murine groups to perform a relevant classification: i) Control (specimens from normal murine lungs) and ii) Bleomycin (specimens from murine bleomycin PF model).

For the classification we used SVM. For better classification results, we employed the following techniques regarding SVM: 1. Feature Extraction, 2. Preprocessing, 3. Kernel Selection, 4. Parameter Tuning, 5. Dimensionality Reduction and 6. Model Evaluation. We used 4 different kernel functions, more specifically: i) Linear SVM, ii) Relu‐based SVM, iii) RBF‐based SVM and iv) Sigmoid SVM. All four kernel function SVM models are employed and tested on the three classification tasks. Further details and explanations of the classification techniques used in this study can be found in the Supporting Information (S1 *In silico* experiments: classification techniques).

### 2.7 Statistical Analysis

All statistical analyses were performed using GraphPad Prism version 10.2.0 (GraphPad Software, CA, USA). Young's modulus values were first normalized within each sample using median centering to reduce intra‐sample variability, and for inter‐group comparisons, values were further normalized to the mean of the control group or transformed using z‐scores when indicated. Outliers were identified and excluded using the ROUT method (Q = 1%). Data are presented as mean ± standard error of the mean (SEM) unless otherwise specified. For comparisons between two groups, unpaired Student's t‐test (for parametric data) or Mann–Whitney U test (for non‐parametric data) was applied. For multiple group comparisons, either one‐way ANOVA with Bonferroni correction (parametric) or the Kruskal–Wallis test followed by Dunn's post hoc test (non‐parametric) was used. Statistical significance was defined as *p* < 0.05, and the following notation was used to indicate significance levels: *p* < 0.05 (*), *p* < 0.01 (**), *p* < 0.001 (***), *p* < 0.0001 (****). Spearman rank correlation was used to assess the relationship between tissue stiffness and collagen content, with correlation strength interpreted as strong (*r* ≥ 0.8). Normality of distributions was evaluated using the Shapiro–Wilk test and visual inspection.

Additional descriptions of materials and methods can be found in the Supporting Information.

## 3 Results

### 3.1 Nanomechanical Properties of Human Lungs with PF

To evaluate the nanomechanical properties of human lungs, specimens from 5 PF patients were obtained while one of the patients (Patient #5) agreed to provide tissue samples from both the fibrotic and the healthy area of the lung. The patient characteristics, including sex, diagnosis, and histological examination results, are described in detail in Τable S1 (Supporting Information).

**Figure 2:**
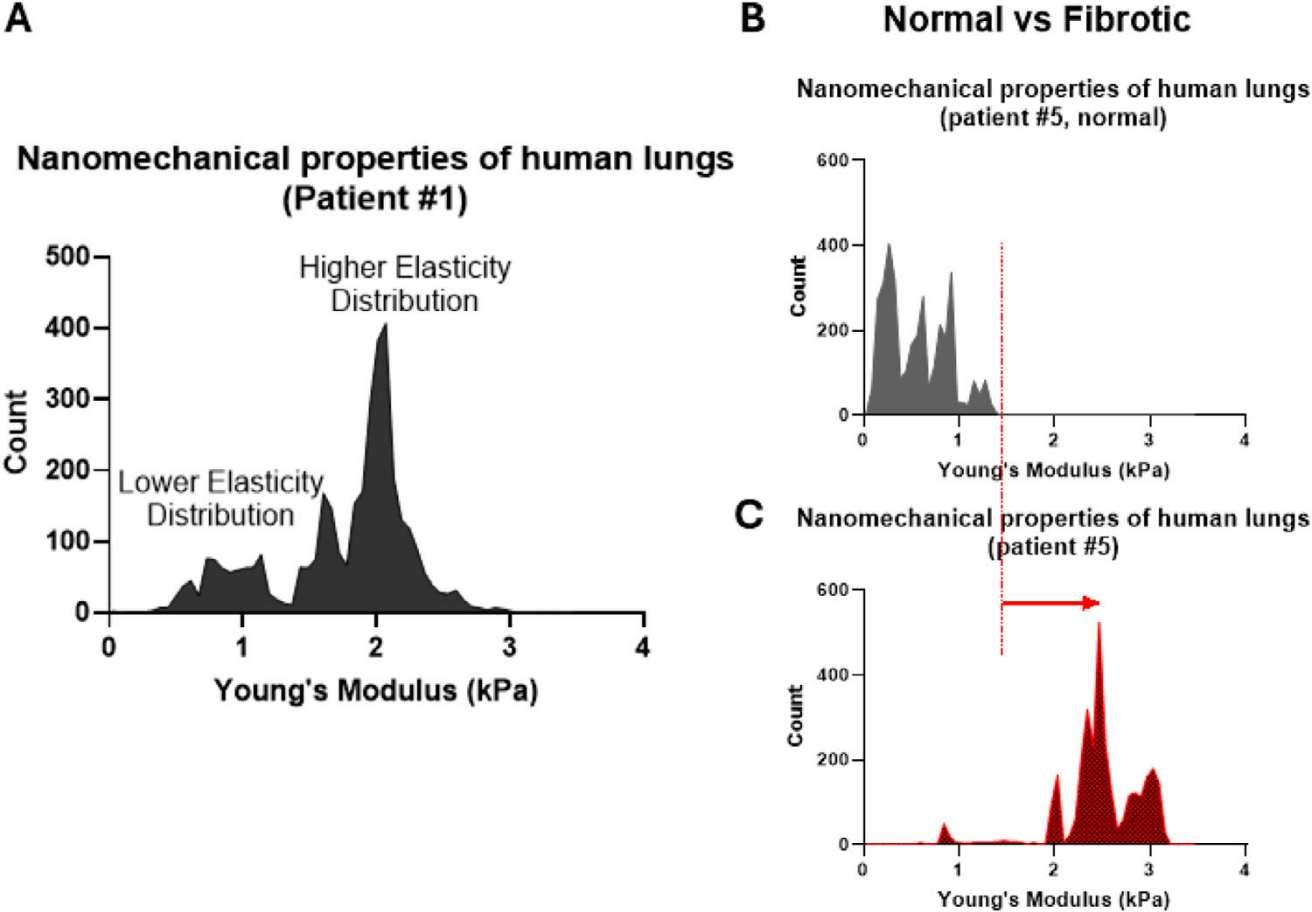
Nanomechanical properties of human lungs. A) Representative elasticity spectrum, combining the characterization of 15 different 20 × 20 µm regions of interest (ROIs) across the entire specimen of a patient with PF (patient #1). The spectrum presents the characteristic lower LEP (likely due to fibroblast softening during PF) and the higher (resulting from collagen overproduction during PF). B,C) Representative elasticity spectra from two different ROIs of a patient with PF (patient #5). The different ROIs are from B) normal and C) fibrotic region of the patient’ lungs. As expected, the elasticity spectrum from the lung region suspected to have fibrosis shows a HEP shifted to higher Young's modulus values compared to the peaks from ROIs of healthy lung tissue.

Using AFM and the proposed protocol, we characterized fresh lung specimens obtained via routine bronchoscopy under liquid conditions within 3 days from tissue harvest (Figure S1, Supporting Information). As shown in **Figure** **2**A, from the obtained spectrum in one PF patient's sample, we can identify two distinct peaks representing two different distributions; a lower elasticity peak/distribution (LEP) and a higher elasticity peak/distribution (HEP). However, these two distributions were not clearly present in all human specimens (Figure S1, Supporting Information), most likely due to inter‐patient heterogeneity. Also, AFM‐based measurements of normalized Young's modulus revealed increased mechanical heterogeneity in fibrotic lung specimens compared to the non‐fibrotic control. Fibrotic tissues (#1–#5) exhibited broader distributions, higher coefficients of variation (up to 857.2%), and wider interquartile ranges than the non‐fibrotic sample (#6), consistent with irregular collagen remodeling. These differences were visualized using box‐and‐whisker plots after ROUT‐based outlier removal and intra‐sample normalization (see Figure S2 and Table S2, Supporting Information).

Nevertheless, the presence of the characteristic peaks (LEP and HEP) in some patients is significant, as a similar pattern has been observed in cancer studies,^[^ ^17^, ^19^, ^20^, ^31^, ^37^ ^]^ where the LEP was associated with reduced stiffness of cancer cells, indicating cell “softening” compared to normal cells.^[^ ^17^, ^38^, ^39^ ^]^ However, similar findings have not been extensively reported in the literature with regard to PF. Interestingly though, we recently demonstrated that Transforming Growth Factor‐β (TGF‐β)‐activated normal human lung (NHL) fibroblasts, which are thought to have acquired a myofibroblast‐like phenotype, are softer than normal fibroblasts.^[^ ^40^ ^]^ Moreover, our in vitro experiments on fibrotic lung cells (LL97A) showed that they are softer than normal lung cells (LL24) (Figure S5A, Supporting Information) and the latter are becoming softer when cultured on stiffer substrates (Figure S5B, Supporting Information). Thus, these findings can collectively justify the observed LEP. Consequently, the activation of NHL fibroblasts through the TGF‐β pathway, which plays a crucial role in PF,^[^ ^41^, ^42^ ^]^ along with tissue stiffening caused by excessive collagen production, further promotes lung cell softening (similarly to what is shown in Figure S5A, Supporting Information where lung cells become softer on stiffer substrates). On the other hand, HEP is associated with collagen overproduction, as evidenced by Collagen I (*COL1*) mRNA expression analysis via real‐time PCR (Figure S4, Supporting Information), leading to increased lung tissue stiffness due to fibrosis.

#### 3.1.1 Differences between Normal and Fibrotic Human Lungs

To examine whether normal and fibrotic human lung specimens exhibit distinct NMFs that can be potentially used as novel biomarkers for diagnosing PF, we proceeded to assess the NMF of patient #5, taking advantage of the fact that we had specimen from both the fibrotic and the healthy area of the lung. Figure 2 B,C show representative elasticity spectra from patient #5. As expected, the elasticity spectrum from the lung region suspected to have fibrosis (Figure 2C) shows a higher elasticity distribution shifted to higher Young's modulus values compared to the peaks from ROIs with healthy lung tissue (Figure 2B). Also, a direct comparison between fibrotic (Sample #5) and non‐fibrotic (Sample #6) lung regions revealed a significant difference in their normalized Young's modulus distributions (*p* = 0.0005, Mann–Whitney U test, Figure S3, Supporting Information). This finding highlights distinct mechanical profiles between fibrotic and healthy tissue areas.

### 3.2 Nanomechanical Properties of Murine Lungs During PF Progression

**Figure 3:**
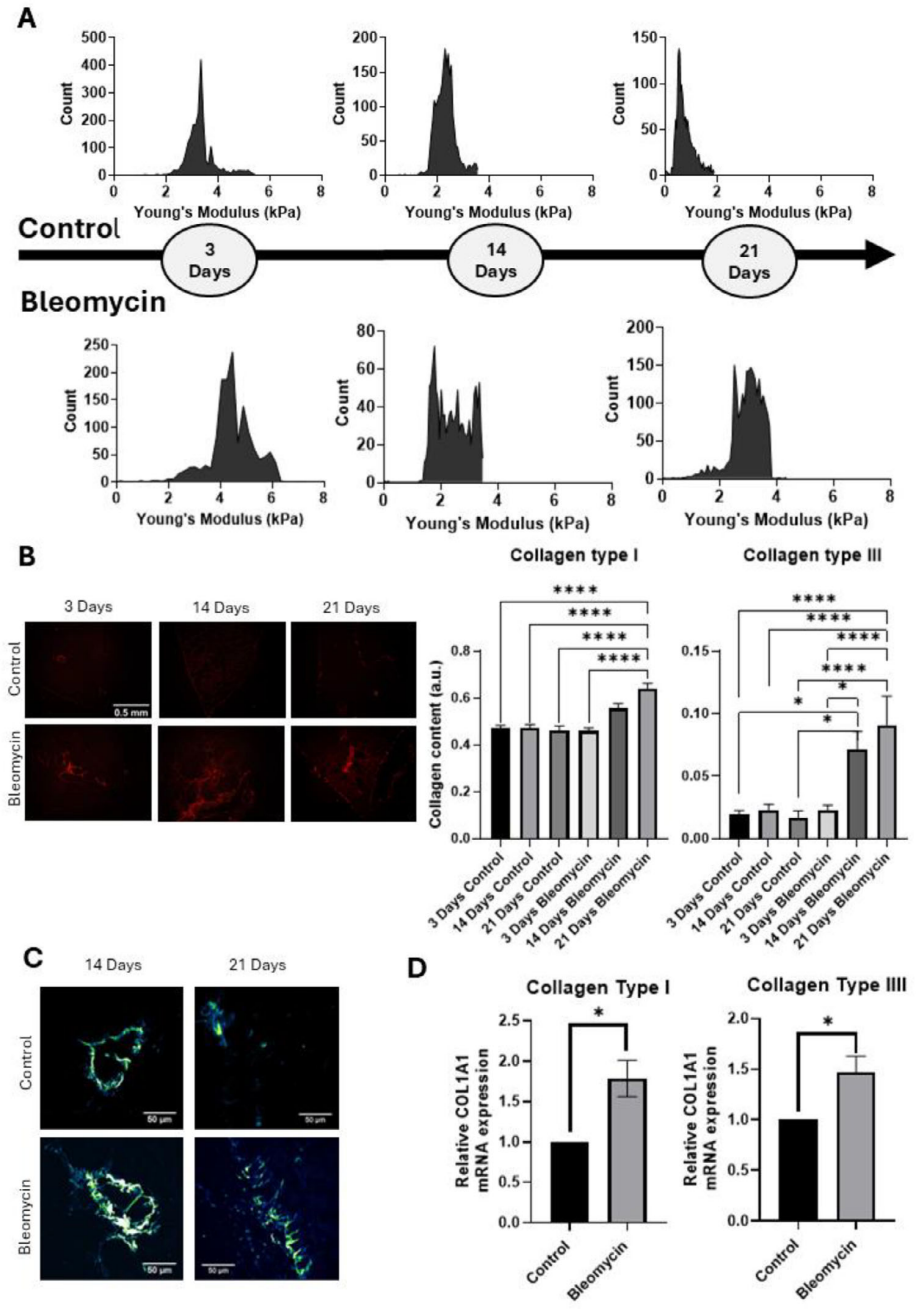
NMFs and collagen content during PF progression. A) Representative Young's modulus spectra at different time points (3‐, 14‐, and 21‐days post bleomycin administration) for the two different groups (control and bleomycin treatment). The spectra show that AFM is sensitive enough to capture nanomechanical alteration during disease progression. B) Representative polarized picrosirius red images and quantification analysis from all the murine specimens for collagen type I and III (scale bar 0.5 mm). For collagen type I, all group comparisons showed statistically significant differences with *p* < 0.0001. For collagen type III, the comparisons between 3 Days Control and 14 Days Bleomycin (*p* = 0.0177), 21 Days Control and 14 Days Bleomycin (*p* = 0.0331), and 3 Days Bleomycin and 14 Days Bleomycin (*p* = 0.0303) were also significant. All other comparisons that are shown in the figure presents significant differences with *p* < 0.0001. Each bar represents the mean ± SEM. C) Representative SHG microscopy images (scale bar 50 µm). D) COL1A1 and COL3A1 mRNA expression from real‐time PCR experiments. Experiments were performed in triplicates and at least two independent experiments were conducted. Quantification of relative mRNA expression was performed using the ΔΔCt method. mRNA expression levels are presented asSEM. Statistically significant differences between control and bleomycin‐treated groups were observed for collagen type I (*p* = 0.0006) and collagen type III (*p* = 0.0034). Each bar represents the mean ± SEM. Statistical significance was indicated as follows: *p < 0.05, **p < 0.01, ***p < 0.001, and ****p < 0.0001.

Subsequently, we set out to investigate nanomechanical properties in a murine PF model. For this purpose, we used bleomycin to induce PF in mice and first investigate any changes in nanomechanical properties at various stages of disease progression. Our results demonstrated that AFM can identify elasticity spectra with distinct NMFs associated with different PF stages (**Figure** **3**A; Figure S6, Supporting Information). The results from the elasticity spectra and the alterations in NMFs were also correlated with alterations in collagen type I and III content during PF, as assessed by polarized microscopy on picrosirius red stained specimens (Figure 3B). The quantification of the images showed a statistically significant increase in collagen type I and III mainly during the chronic fibrosis stage (Figure 3B), while a gradual increase was observed during PF progression from day 3 to day 21 post bleomycin administration. This increase in collagen content was not observed in control samples, showing that the observed alterations in collagen content were not a result of aging, but a consequence of PF progression.

To further explore the relationship between collagen deposition and tissue stiffening during fibrosis progression, we performed a correlation analysis between collagen content (quantified from picrosirius red‐stained lung sections) and Young's modulus values measured with AFM (Section S4.2, Supporting Information). This analysis was conducted separately for each experimental time point (i.e., 3, 14, and 21 days post‐bleomycin treatment). While no correlation was detected at day 3 (inflammation phase), a moderate but non‐significant trend emerged on day 14. On day 21, that corresponds to the ECM remodeling phase, a strong and statistically significant positive correlation was observed (*r* = 0.886, *p* = 0.0333), suggesting that collagen accumulation substantially contributes to increased lung stiffness at later fibrotic stages. Full statistical details and correlation plots are presented in Figure S7 (Supporting Information).

Additionally, in order to facilitate meaningful comparison between species, we applied z‐score normalization to AFM‐derived Young's modulus values from fibrotic human lung samples and fibrotic murine lungs at Days 14 and 21 post‐bleomycin treatment. This analysis, detailed in Section S4.3 (Supporting Information), showed that Day 21 murine samples exhibited mechanical properties more closely aligned to human fibrotic tissue, compared to Day 14 samples (Figure S8). Our findings support the use of late‐stage murine fibrosis models as more accurate mechanical analogs of the human disease.

As collagen is an ideal emitter of strong SHG signals due to its non‐centrosymmetric structure,^[^ ^43^, ^44^ ^]^ SHG imaging microscopy was employed as a high‐resolution, label‐free method for non‐invasively detecting collagen organization and distribution in murine lung specimens (Figure 3C). The representative SHG images demonstrate a significant increase in collagen signals in bleomycin‐treated mice compared to control. Additionally, both picrosirius red staining and SHG imaging revealed increased collagen deposition during the chronic fibrosis stage, which was further validated by real‐time PCR experiments. The results showed a statistically significant increase, both at the protein (Figure 3B,C) and the mRNA level (Figure 3D), in collagen types I and III during the chronic fibrosis stage.

The collective findings suggest that AFM‐based NMFs serve as a robust tool for comprehensively assessing PF progression, shedding light on both the disease‐specific alterations and age‐related effects on tissue mechanics.

### 3.3 Nanomechanical Properties of Lungs as a Possible Diagnostic Biomarker

To further substantiate the application of NMFs as potential biomarkers and to validate our experimental findings, we conducted a series of *in silico* experiments using three types of datasets; i) optical microscopy images from picrosirius red‐stained tissues, ii) AFM‐derived Young's modulus values from lung tissues, and iii) a combined dataset of optical microscopy images and AFM‐based measurements. For each dataset, we classified the specimens into two categories; i) control (i.e., specimens from normal lungs) and ii) bleomycin‐treated specimens from the bleomycin model of PF. In total, we collected data from 16 control and 17 bleomycin‐treated mice. To increase the amount of training data and to ensure robust cross‐validation, we partitioned the available data into multiple non‐overlapping subsets by segmenting the images and AFM data into smaller, independent samples. This data augmentation approach improved the statistical power of our analysis and enhanced the generalization capability of the classifier. For classification we used SVMs.^[^ ^45^, ^46^ ^]^ The SVM classifier was chosen due to its proven effectiveness in image classification tasks, especially for small to medium‐sized datasets with relatively simple features,^[^ ^47^ ^]^ as was the case with our optical microscopy images.

Our primary goal was to achieve the highest possible classification accuracy by combining both optical microscopy and AFM data. Specifically, we aimed for the classifier to leverage the richer feature set to improve the decision‐making process.

#### 3.3.1 Labeling Criteria

Each data segment (image region or AFM measurement) was labeled based on its original specimen's known status as either control (healthy) or bleomycin‐treated (fibrotic).

#### 3.3.2 Validation Strategy

For all classification tasks, we employed k‐fold cross‐validation (k = 20) to ensure unbiased performance estimation and to prevent overfitting. In each fold, the data were randomly partitioned into training and testing sets, ensuring that data segments from the same specimen were not split across training and testing sets within a fold, preserving data independence.

#### 3.3.3 Performance Metrics

Reported accuracies always refer to the average testing accuracy obtained during cross‐validation, unless otherwise stated.

The first step in the pipeline was feature extraction.^[^ ^48^, ^49^ ^]^ Common techniques for feature extraction in image classification include Histogram of Oriented Gradients (HOG), Scale‐Invariant Feature Transform (SIFT), Speeded Up Robust Features (SURF), and Local Binary Patterns (LBP).^[^ ^50^, ^51^ ^]^ To further improve SVM performance, we applied preprocessing techniques, such as normalization and cropping. SVMs map the input data into a higher‐dimensional feature space using a kernel function, making the data linearly separable. The choice of kernel is crucial for achieving optimal accuracy, and we tested four types: Linear SVM, Relu‐based SVM, RBF‐based SVM, and Sigmoid SVM.^[^ ^52^ ^]^ Hyperparameters, including the regularization parameter (C) and kernel parameters (e.g., gamma for RBF), were optimized via grid search during cross‐validation.

We began the processing with the optical microscopy data and each kernel was evaluated across three classification tasks. All the results are summarized in **Table** **1** and correspond to the average testing accuracy from cross‐validation. The Linear SVM yielded the lowest accuracy, ranging from 69–82%, while the Sigmoid SVM provided the best performance with 83.5‐87% (Table 1, Optical Microscopy Images column).

**Table 1:** Summary of the in silico studies that were performed using three different data sets. The classification task: control and bleomycin treated.

Data	Optical Microscopy Images	AFM Data	Optical Microscopy Images + AFM Data
Biomarker	Optical Biomarker	Nanomechanical Biomarker	MechanoOptical Biomarker
**Model**	**Accuracy**		
Linear SVM	69‐82%	70.5‐84%%	79.5‐91%
Relu‐based SVM	77‐84%	78.5‐86%	86‐93.5%
RBF‐based SVM	81‐86%	83‐88%	94‐98%
Sigmoid SVM	83.5‐87%	85‐89.5%	95.5‐98.5%

Following the same process, we classified the AFM data. Due to the simpler dimensionality and well‐defined features, this dataset yielded higher accuracy compared to the images (Table 1, AFM data column). Specifically, the Linear SVM achieved testing accuracies of 70.5–84%, while the Sigmoid SVM reached 85–89.5%.

Combining the two datasets further improved classification accuracy thanks to the larger and more diverse feature set (Table 1, Optical Microscopy Images + AFM Data column). Using both data types, the Linear SVM achieved 79.5–91% accuracy, and the Sigmoid SVM yielded the best results with accuracy of 95.5–98.5%. These results demonstrate that combining AFM and optical microscopy images using the appropriate model (in this case, the Sigmoid SVM) can effectively distinguish normal and fibrotic lung tissue. Our approach supports the development of a novel MechanoOptical biomarker for PF diagnosis.

Finally, to externally validate the model, we tested it on the small human lung dataset from the experiments presented in Section 3.1 (including the 6 human specimens). The classifier correclty identified all five fibrotic patients across all trials and kernels. For the single healthy patient, the classifier showed a slightly lower accuracy of 72% in one fold, indicating occasional misclassification as fibrotic. Despite the limited sample size, this external validation highlights the potential for real‐world clinical application.

### 3.4 Nanomechanical Properties of Murine Lungs for Monitoring Treatment

Next, in order to evaluate the potential of NMFs in monitoring treatment response, we examined the sensitivity of AFM elasticity spectra in detecting changes in lung nanomechanics during treatment with pirfenidone, an approved drug for IPF,^[^ ^5^, ^53^, ^54^, ^55^ ^]^ that has been also used for TME normalization in cancer models.^[^ ^26^, ^56^ ^]^

#### 3.4.1 NMFs During the Treatment within the “Therapeutic” Period

**Figure 4:**
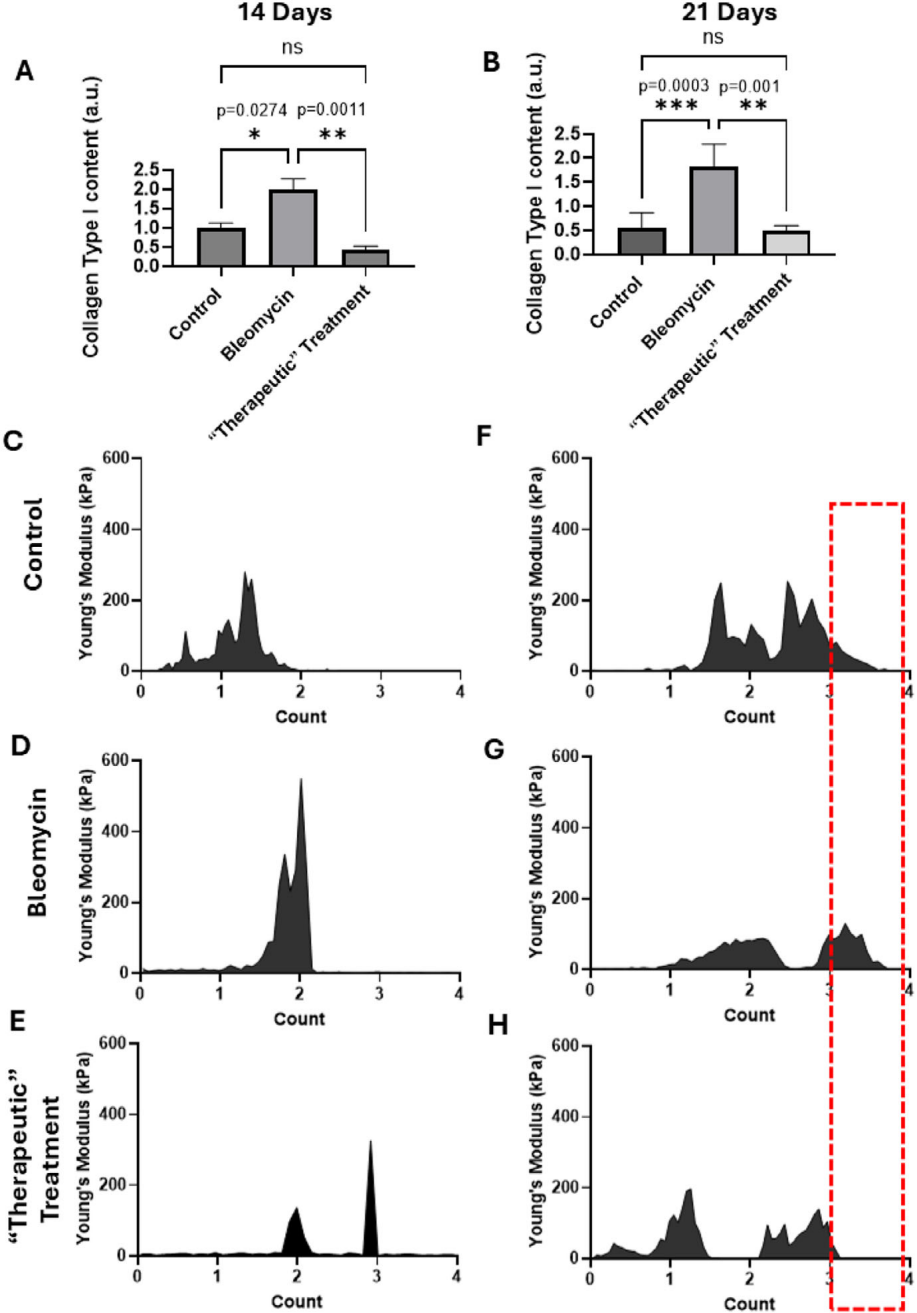
NMFs and Collagen Quantification in Lungs Collected 14‐ and 21‐Days Post‐Bleomycin Administration during the “therapeutic treatment”. A,B) Quantification of collagen content from picrosirius red images, indicating higher collagen levels in the bleomycin group and a significant reduction following Pirfenidone treatment. Four (4) specimens per group (A) and 8 control specimens, 4 Bleomycin and 4 “therapeutic treatment” (B). Each bar represents the mean ± SEM. Statistical significance was indicated as follows: *p < 0.05, **p < 0.01, ***p < 0.001, and ****p < 0.0001. C–E) and F–H) Elasticity spectra illustrating alterations in elasticity peaks, demonstrating the impact of Pirfenidone treatment on lung tissue mechanics. The red dot square marks changes in the higher elasticity distribution highlighting increased elasticity values in the bleomycin (fibrotic) model.

Initially, we assessed NMFs during the “therapeutic” period of PF, spanning from 7 to 21 days post‐bleomycin administration (Figure 1). To that regard, lung tissues were collected 14 days post‐administration during the transition phase from inflammation to active fibrosis and 21 days post‐administration during the chronic fibrosis stage (**Figure** **4**).

Tissue specimens were stained with picrosirius red (representative images are shown in Figure S9A, Supporting Information). The quantification of collagen content using picrosirius red staining (Figure 4A,B) indicated that, as expected, the bleomycin‐treated group, exhibited higher collagen levels that were significantly reduced in mice receiving treatment with Pirfenidone. Notably, elasticity spectra demonstrated distinct alterations in elasticity peaks both during the transition phase from inflammation to active fibrosis (Figure 4C–E) and during the the chronic fibrosis stage (Figure 4F–H). Especially during the last stage, the elasticity spectra showed a more pronounced removal of high elasticity distribution due to Pirfenidone treatment (see red box in Figure 4F–H).

Furthermore, to enhance the robustness of our findings, we conducted supplementary experiments using a small cohort of male mice (data now presented in Section S4.4 and Figure S10, Supporting Information). The results demonstrated that male mice exhibited NMFs similar to those observed in female mice (Figure S10A, Supporting Information). Following bleomycin treatment, a clear shift in NMFs and an increase in Young's modulus were detected (Figure S10B, Supporting Information). Notably, treatment with pirfenidone during the “therapeutic phase” led to changes in both elastic peak distributions and overall stiffness (Figure S10C,D, Supporting Information). These mechanical alterations were accompanied by changes in collagen content, as shown by quantitative analysis of picrosirius red‐stained sections (Figure S10E,F, Supporting Information).

#### 3.4.2 NMFs During the Treatment within the “Prevention” Period

**Figure 5:**
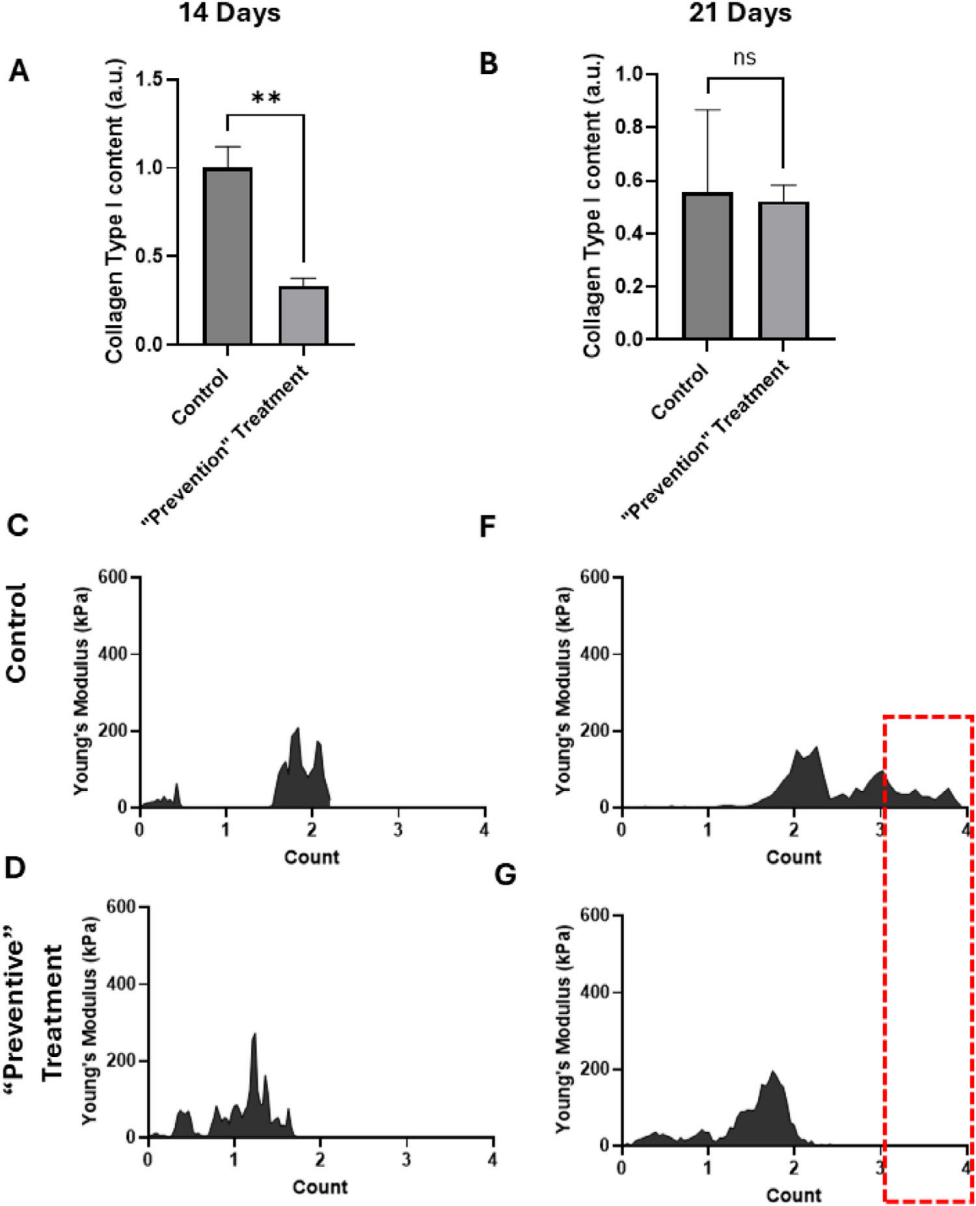
NMFs and Collagen Quantification in Lungs Collected 14 and 21 Days Post‐Bleomycin Administration post “Prevention Treatment”. A,B) Quantification of collagen content from picrosirius red images, indicating higher collagen levels in the bleomycin group and a significant reduction following Pirfenidone treatment (8 specimens for control and 4 specimens for treatment groups). Each bar represents the mean ± SEM. Statistical significance was indicated as follows: *p < 0.05, **p < 0.01, ***p < 0.001, and ****p < 0.0001. C–E) and F–H) Elasticity spectra illustrating alterations in elasticity peaks, demonstrating the impact of Pirfenidone treatment on lung tissue mechanics. The red dot square indicates changes in the higher elasticity distribution highlighting decreased elasticity values during pirfenidone treatment in the “preventive” window.

Next, we set out to study the NMFs of PF following Pirfenidone treatment during the “preventive” period, also known as early treatment (**Figure** **5**). Lung specimens were collected at two different time points; 14 and 21 days post‐ bleomycin administration.

The specimens were stained with picrosirious red staining and SHG microscopy was also employed (Figure S9, Supporting Information). The quantitative results showed a significant decrease in collagen content (Figure 5A) in the treated lungs compared to control lungs after 14 days. However, there was no significant alteration after 21 days (Figure 5B). The NMFs, from the elasticity spectra are in aggrement with the collagen alterations (Figure 5C–G), and especially 21 days post treatment the higher elasticity spectrum is clearly absent. Thus, the results indicate that preventing treatment can prevent fibrosis and the AFM‐based NMFs can indeed monitor the relevant alterations.

## 4 Discussion

Mechanobiology approaches are ideal for studying diseases that involve changes in the tissue structure and mechanics, including those that occur in PF. Therefore, they can offer significant benefits in addressing limitations related to PF management, such as lack of specific diagnostic biomarkers for classification of disease staging and for treatment monitoring. Here, we used AFM to identify unique NMFs to characterize fibrosis stages and monitor treatment response. While macroscopic techniques such as rotational rheometry or ultrasound elastography can effectively capture bulk mechanical changes in fibrotic tissues, they lack the resolution to detect cellular and microscale matrix alterations. AFM on the other hand, uniquely enables simultaneous assessment of cell mechanics and collagen‐driven microenvironmental changes, which is critical for identifying early, localized mechanobiological signatures of fibrosis.

The study focused primarily on murine models of PF to establish AFM‐based NMFs as a sensitive and label‐free tool for assessing disease progression and treatment response. Human lung specimens were included to serve as proof‐of‐concept, demonstrating the translational potential of this approach. Our goal was to investigate whether AFM‐derived mechanical signatures could reflect the pathological changes observed in fibrotic lung tissue—particularly alterations in stiffness linked to ECM remodeling. Rather than proposing AFM as a replacement for traditional histological or molecular assays, we present it as a complementary method that captures biomechanical features often overlooked but central to the pathophysiology of PF. The consistency between murine and human data underscores the potential use of NMFs as diagnostic or monitoring biomarkers in clinical settings.

AFM analysis of human lung specimens from PF patients revealed distinct nanomechanical profiles, with some samples exhibiting characteristic dual peaks in elasticity spectra—namely, a lower elasticity peak (LEP) and a higher elasticity peak (HEP). Overall, fibrotic tissues showed markedly increased mechanical heterogeneity compared to non‐fibrotic controls, reflecting the irregular and variable nature of ECM remodeling in PF. Although the sample size of human cases was limited, the reproducible differences in nanomechanical profiles support the potential use of AFM‐based diagnostics in PF. It should be noted that the limited number of human samples was due to patient limited availability and willingness to participate. Despite this limitation, our study used these samples as proof of principle that AFM can be employed to characterize NMFs in PF in humans. Future studies with larger, stratified cohorts will be essential for a more robust clinical validation.

One of the most noteworthy findings of our work is the ability of AFM to detect subtle changes in the nanomechanical properties of lung tissues as fibrosis progresses. The identification of distinct peaks in Young's modulus spectra—specifically, LEP and HEP—provides a novel approach to characterize the fibrotic state. The LEP, associated with fibroblast softening (which was confirmed with our in vitro experiments), and the HEP, linked to collagen overproduction, were consistently observed across different stages of fibrosis both in human patients and murine PF models. Our results suggest that AFM‐derived NMFs are sensitive enough to capture the mechanical alterations that occur during fibrosis progression, making them a promising tool for disease staging. Notably, our findings in human patient samples are in accordance with the findings from in vivo experiments conducted on the bleomycin‐induced PF model, which highlights the potential of AFM‐based nanomechanical signatures as diagnostic PF biomarkers. The ability to detect distinct NMFs associated with fibrotic tissue could lead to earlier and more accurate diagnosis, ultimately improving therapeutic outcome. Furthermore, the application of AFM technology in this context may pave the way for new research into the mechanical properties of fibrotic tissues, offering insights into disease progression and potential therapeutic targets.

The observed NMFs, particularly the LEP, were further supported by in vitro studies, which showed that lung fibroblasts become softer and more elongated when cultured on stiffer substrates—mirroring the mechanical environment present during PF progression. While increased cells’ stiffness is often linked to cytoskeletal reinforcement, our findings show that fibroblasts become softer on stiffer substrates, an effect consistent with reports of stiffness‐induced softening (“stiffness‐matching”).^[^ ^57^ ^]^ This response likely reflects a mechanical adaptation of the cells in order to preserve cellular tension balance in excessively rigid environments.^[^ ^57^, ^58^ ^]^ Additionally, recent studies have demonstrated that stiff matrices can lead to either fibroblast stiffening or softening, depending on several factors such as duration of exposure, cytoskeletal remodeling, and the involvement of key mechanotransduction pathways.^[^ ^40^, ^57^, ^59^, ^60^, ^61^, ^62^, ^63^ ^]^ For instance, it has been shown that fibroblasts become stiffer when cultured on stiff substrates. Indeed, AFM measurements showed that fibroblasts grown on fibronectin‐coated polyacrylamide gels exhibit a modulus that closely matches or slightly underestimates the stiffness of the substrate.^[^ ^57^ ^]^ Similarly, traction force microscopy and imaging studies demonstrated that increased substrate rigidity leads to larger traction forces, enhanced F‐actin organization and stress fiber formation, increased cell spreading, and nuclear elongation.^[^ ^63^ ^]^ However, other studies are in agreement with our results and have reported that increasing substrate stiffness causes lung fibroblasts to adopt a more spindle‐shaped, spread morphology along with elevated expression of α‐smooth muscle actin (α‐SMA) and enhanced migration.^[^ ^59^ ^]^ Fibroblasts exposed to matrices mimicking the elastic modulus of fibrotic tissue also undergo differentiation into a contractile myofibroblast phenotype.^[^ ^60^ ^]^ Moreover, our findings align with previous reports showing that fibrotic stiffness induces the expression of genes and secreted factors associated with fibrotic responses as well as the senescence‐associated secretory phenotype.^[^ ^61^ ^]^ These contrasting findings indicate that fibroblast responses to substrate stiffness are context‐dependent, and further research is needed to draw definitive conclusions about their mechanobiological behavior across different stiffness environments. In relation to the observed decrease in circularity on stiffer substrates (Figure 3D), elongation and stress fiber formation are often associated with increased cellular contractility. However, this does not necessarily result in increased stiffness at the nanoscale. Spatial heterogeneity in cytoskeletal tension and adaptive responses may still lead to an overall softening of the cell. This is in accordance with pertinent studies in cancer, where highly metastatic cells have shown to be more elongated and mechanically softer than their non‐malignant counterparts, suggesting a link between reduced stiffness and enhanced invasive potential.^[^ ^31^, ^39^, ^64^, ^65^, ^66^ ^]^ Similarly, our finding that lung fibroblasts become more elongated and softer when cultured on stiffer, fibrotic‐like substrates is consistent with these observations. Further studies are needed to explore whether this mechanically adaptive phenotype affects key fibroblast functions, such as migration and contributes to fibrotic tissue remodeling.

Moreover, our findings indicate that these nanomechanical signatures can be used not only for diagnosis but also for monitoring treatment efficacy. The observed changes in NMFs following treatment with the anti‐fibrotic drug Pirfenidone underscore the potential of AFM‐based biomarkers to predict and evaluate treatment responses. Our results were confirmed both in female and male PF murine models. The reduction in collagen content and the corresponding alterations in elasticity spectra after Pirfenidone treatment suggest that these nanomechanical measurements can provide real‐time insights into the therapeutic effects, which is crucial for the development of personalized treatment strategies. The majority of experiments were conducted in female murine models; however, key findings were also confirmed in a small cohort of male mice. These additional experiments demonstrated similar NMFs between sexes, with bleomycin treatment causing a shift in elasticity profiles and increased tissue stiffness. Pirfenidone treatment during the therapeutic phase led to notable changes in both elasticity distributions and collagen content, further validating the consistency and robustness of our results across sexes.

Furthermore, the in silico classification experiments provided robust validation for the potential use of AFM‐based NMFs as novel biomarkers for PF. By employing SVM algorithms on three distinct datasets ‐optical microscopy images, AFM‐based Young's modulus measurements, and a combined dataset‐ we were able to effectively differentiate between normal and fibrotic lung tissues. Notably, the combination of AFM data with optical microscopy images, particularly when using a Sigmoid SVM model, demonstrated high accuracy in classifying the specimens, highlighting the synergistic value of integrating mechanical and optical data. These findings highlight the promise of AFM‐derived nanomechanical signatures as a complementary diagnostic tool, that could render them ideal MechanoOptical biomarkers in clinical settings for more accurate and personalized PF diagnosis and monitoring.

Interestingly, similar alterations in Young's modulus spectra have been observed during cancer progression. The fact that the mechanical properties of tumors are increasingly recognized as critical factors in disease progression and treatment response,^[^ ^31^, ^37^, ^67^, ^68^ ^]^ suggests a broader applicability of the presented AFM‐based approach. Thus, AFM‐based NMFs could have multiple applications, potentially leading to common diagnostic and therapeutic approaches across different pathological conditions, such as fibrosis and cancer. The similarities between PF and cancer in terms of mechanical alterations also highlight the potential of repurposing TME normalization agents, traditionally used in cancer treatment, for managing fibrosis. Drugs such as Pirfenidone, approved for PF, have also shown promise in altering tumor mechanics^[^ ^26^, ^56^ ^]^ and could be equally effective in modulating the fibrotic microenvironment in both PF and cancer, thereby improving treatment outcomes.

This is the first time that AFM‐based NMFs have been identified as potential biomarkers for PF, opening up new avenues for research with obvious clinical relevance. These biomarkers could significantly enhance the diagnostic precision and efficacy of treatment, addressing the urgent need for developing personalized approaches in managing PF. Moreover, the integration of AFM‐based data with traditional histopathological and gene expression analyses offers a comprehensive method for characterizing the mechanical state of fibrotic tissues, which could be easily transferred to clinical settings. However, it should be noted that the use of NMFs as potential biomarkers require extensive studies before being translated into clinical practice. A large amount of data, mainly from human patients, must be collected to support their application. Although NMFs might not serve as stand‐alone diagnostic or prognostic biomarkers, could be used alongside existing biomarkers to develop personalized disease signatures, promoting tailored treatments.

The findings of this study can open several promising directions for future research and clinical translation. AFM‐based nanomechanical profiling could be further explored as a minimally invasive diagnostic or prognostic biomarker in fibrotic diseases, potentially through its adaptation to biopsy‐based or endoscopic sampling workflows. Although our findings demonstrate clear nanomechanical alterations in fibrotic lung tissue, future studies will be required to determine the specificity of these mechanical biomarkers in comparison to other lung pathologies, such as cancer and inflammation. Comparing these findings with other lung diseases will be important to specifically understand how these mechanomarkers truly behave and how useful they could be in real clinical settings. Moreover, the integration of mechanical signatures with molecular and imaging data could lead to more comprehensive, multimodal fibrosis staging systems. Finally, the demonstrated ability of NMFs to monitor therapeutic response suggests that AFM could serve as a useful tool in drug development pipelines, enabling earlier assessment of treatment efficacy and personalized therapy optimization. Future studies integrating nanomechanical profiling with genomic, histopathological, and biochemical analyses could provide a more comprehensive understanding of disease progression, support the development of robust, multi‐modal diagnostic tools and promote personalized treatments.

In conclusion, this study provides strong evidence that AFM‐derived nanomechanical signatures can serve as reliable biomarkers for the diagnosis and treatment monitoring of PF. In fact, the ability of these biomarkers to predict treatment responses and their potential applicability across different diseases emphasize their value in advancing personalized medicine.

## Conflict of Interest

The authors declare no conflict of interest.

## Author Contributions

All authors contributed to the manuscript writing and have given approval for the final version of the manuscript. A.S. performed data curation. A.S., K.P., and V.A. performed formal analysis. A.S. performed funding acquisition. A.S., K.P., F.M., M.M., G.F. performed investigation. A.S. performed methodology. A.S., G.F., A.Z., P.P.S., V.G., and T.S. performed resources. K.P., V.A., C.V., S.V.K., and P.P.S. performed software. A.S., T.S. performed Supervision. A.S. performed visualization. A.S., K.P., V.A., F.M., C.V., M.M., G.F., A.Z., S.V.K., P.P.S., V.G., and T.S. wrote original draft. A.S., F.M., V.G., and T.S. wrote and edited.

## Supporting information



Supporting Information

## Data Availability

The data that support the findings of this study are available from the corresponding author upon reasonable request.
