# Supplementary material for: Atomic Force Microscopy‐Based Nanomechanical Signatures for Staging Classification and Drug Response in Pulmonary Fibrosis
Source: Small. 2025 Sep 27;21(44):e04526. doi: 10.1002/smll.202504526 (PMC12590530; doi:10.1002/smll.202504526)
Supplement: Supplementary file 1 — Supporting Information [file SMLL-21-e04526-s001.doc]

# Supplementary Material

## **S1. *In silico* experiments: classification techniques**

Starting from a classification task that involves image classification (optical microscopy image), we needed to choose the appropriate algorithm or machine learning model. Our main criteria were high classification accuracy, reduced computational complexity, high speed, and the complexity of the implemented model. Some popular and effective classifiers for image classification include:

1. Convolutional Neural Networks (CNNs): Ability to automatically learn hierarchical features
2. Transfer Learning Models: Models pre-trained on large-scale datasets
3. Support Vector Machines (SVMs): SVMs are effective for small to medium-sized datasets with relatively simple image features.
4. Random Forests: For structured image data.
5. Gradient Boosting Machines: For handcrafted features or as ensemble methods.
6. Deep Learning Architectures: Higher accuracy in video classification.

Based on our main criteria we finally selected Support Vector Machine (SVM). SVMs work well when the number of features is less than the number of samples, and they can handle high-dimensional feature spaces efficiently.

For better classification result we employed the following techniques regarding SVM:

1. Feature Extraction: SVMs work best with well-defined and informative features. We extracted relevant features before training the SVM. Common techniques used for feature extraction in image classification include Histogram of Oriented Gradients (HOG), Scale-Invariant Feature Transform (SIFT), Speeded Up Robust Features (SURF), and Local Binary Patterns (LBP).
2. Preprocessing: Preprocessing techniques were employed such as normalization, resizing, and cropping which improved the performance of SVMs by making the input data more uniform and reducing noise or irrelevant information.
3. Kernel Selection: SVMs use a kernel function to map the input data into a higher-dimensional feature space where it can be linearly separable. Common kernel functions include linear, Relu-based, radial basis function (RBF), and sigmoid. We made experiments with different kernel functions to find the one that works best for our dataset.
4. Parameter Tuning: SVMs have hyperparameters that need to be tuned for optimal performance. These include the regularization parameter (C) and the kernel parameters (e.g., gamma for RBF kernel). Techniques like grid search and random search were used to find the optimal hyperparameters through cross-validation.
5. Dimensionality Reduction: We have checked if this technique will upgrade the classification accuracy. If you have a high-dimensional feature space, dimensionality reduction techniques like Principal Component Analysis (PCA) or Linear Discriminant Analysis (LDA) can be used to reduce the dimensionality of the feature space while preserving as much information as possible. We observed that in this specific task this technique does not affect the accuracy.
6. Model Evaluation: Evaluate the performance of the SVM classifier using appropriate metrics such as accuracy, precision, recall, F1-score, and ROC curves. Additionally, we considered by using techniques like cross-validation to assess the generalization performance of the model.

For simplicity, we employed the same techniques and models in AFM-based measurments (we made the necessary modifications based on the nature of the data).

We used 4 different kernel function, more specifically:

- Linear Support Vector Machines (SVM): Useful for linearly separable data. It’s computationally less intensive and can be a good starting point for SVMs. Simple, fast to compute, and effective when the number of features is large relative to the number of samples.
- Relu-based SVM: While ReLU (Rectified Linear Unit) is typically used in neural networks, a ReLU-based kernel for SVMs can be constructed. One approach is to use a piecewise linear approximation. This kernel function behaves similarly to the ReLU activation function by zeroing out negative values. Useful when trying to leverage ReLU-like behavior in SVMs, particularly when dealing with sparse data or features with many zeros. Can capture non-linearities similar to RBF but with a piecewise linear behavior that might be advantageous in certain types of data distributions.
- RBF-based SVM: Effective for non-linear data where the relationship between the class labels and attributes is not linear. The gamma parameter (γ) controls the influence of individual training examples. A low gamma means a far influence, and a high gamma means a close influence. Can handle complex relationships in the data, works well in most scenarios.
- Sigmoid SVM: Similar to neural networks, it maps the data into a sigmoid space. It’s not as commonly used as linear or RBF but can be useful in specific cases. Alpha (α) and constant term (c) need to be tuned (neural network’s equation parameters). Can model complex relationships, though it might not perform as well as RBF in many cases.

Selecting and tuning the kernel function, can significantly improve the performance of SVMs in different classification tasks. All four kernel function SVM models are employed and tested on the three classification tasks. We have implemented for all cases both the software-based model and the hardware-friendly approximation one.

## ***S2. Ex vivo experiments with human specimens***

## **S2.1 Patient Characteristics**

**Supplementary Table 1:** **Patient Characteristics and Specimen Details**. Summary of patient sex, diagnoses, and histological examination results. Specimens were freshly characterized using Atomic Force Microscopy (AFM) to investigate nanomechanical properties.

|  | **Gender** | **Diagnosis** | **Histological examination** | **Evidence of malignancy** |
| --- | --- | --- | --- | --- |
| #1 | Female | Hypersensitivity pneumonitis | Airway centered (peribronchiolar) changes consisting of diffuse fibrotic changes with lymphocytic infiltration with granulomas and interstitial giant cells.  The findings are most consistent with Hypersensitivity pneumonitis | No |
| #2 | Male | Mild interstitial fibrosis | Lung parenchyma in wich there is a mild loss of alveolar architecture. There is mild interstitial fibrosis associated with a mild non specific chronic inflammatory cell infiltrate. There is no vasculitis or evidence of UIP | No |
| #3 | Female | Mild interstitial fibrosis | Lung parenchyma in wich there is a moderrate loss of alveolar architecture. There is also mild interstitial fibrosis associated with a mild non specific chronic inflammatory cell infiltrate. | No |
| #4 | Male | Mild to  moderate non-specific chronic inflammatory cell infiltrate | Alveolated and non alveolated lung biopsy from bronchial wall infiltrated by mild to moderate non-specific chronic inflammatory cell infiltrate. Staining distribution of all immunohistochemical markers CK7, CK5/6, TTF1, P63  shows normal immunoreactivity to the bronchial epithelium. | No |
| #5^*^ | Female | Mild interstitial  fibrosis | Interstitial lymphoplasmacytic infiltrates in a uniform distribution with mild interstitial fibrosis. | No |

*^*^ (patient #5 provided tissue from both the suspicious pathological area and a healthy area.*

## **S2.2 Elasticity spectra from human specimens**


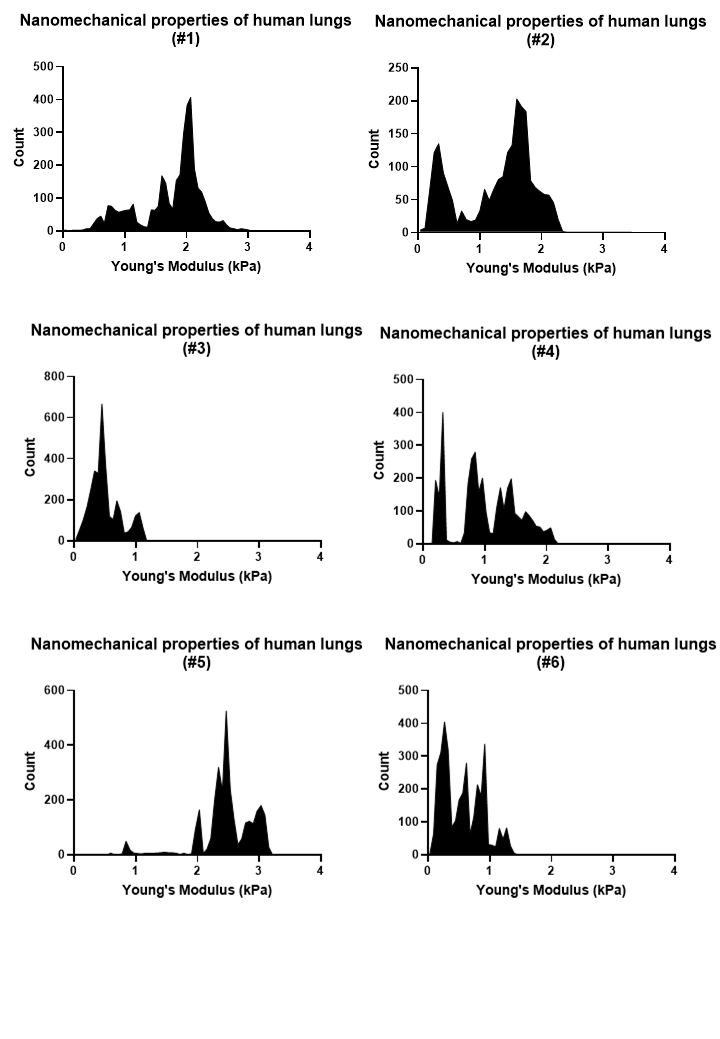


**Supplementary Figure 1: Elasticity spectra of all human specimens.**

**S2.3 Distribution of Normalized Nanomechanical Measurements in Human Lung Specimens**

Normalized Young’s modulus values were obtained from AFM measurements performed in six human lung tissue specimens. The raw Young’s modulus values were first refined using ROUT outlier detection (Q = 1%), then normalized within each specimen by subtracting the median value to reduce intra-sample variability. This normalization enabled direct comparison of nanomechanical signatures across specimens. Box-and-whisker plots (min–max with individual points) were used to visualize intra-sample distribution and heterogeneity (Supplementary Figure 2).

***Supplementary Figure 2: Box-and-whisker plots showing the distribution of normalized Young’s modulus values for each human lung specimen (#1 to -#6).*** *Each point represents a single AFM measurement. Boxes indicate the interquartile range (IQR), lines indicate median values, while the % values correspond to the Coefficient of Variation (CV) values.*

***Mechanical Heterogeneity***: The specimens from fibrotic regions (#1–#5) showed consistently broader distributions of normalized Young’s modulus values compared to the specimen that was collected from a non-fibrotic area and served as control (#6), reflected by their higher coefficients of variation (up to 857.2%), wider interquartile ranges, and greater minimum–maximum spans. While the non-fibrotic specimen (#6) also exhibited mechanical variability (CV = 610.2%), the fibrotic regions tended to show more extreme values and asymmetry, consistent with irregular collagen deposition and tissue remodeling. The high coefficients of variation observed in AFM-derived Young’s modulus values are consistent with the known mechanical heterogeneity of fibrotic lung tissue[[1]](#footnote-2). Similar levels of variability are expected in biological AFM studies, particularly in tissues with uneven extracellular matrix remodeling. By normalizing the data and applying rigorous outlier detection, we ensured that this variability reflects biological differences rather than measurement error.

***Comparison Between Fibrotic (#5) and Non-Fibrotic (#6) Tissue Regions:*** Young’s modulus values, normalized per sample, were compared between the fibrotic area (Sample #5) and the non-fibrotic area (Sample #6) from the same patient (Supplementary Figure 3). The Mann–Whitney U test revealed a statistically significant difference (*p* = 0.0005, two-tailed), indicating distinct mechanical profiles between the two regions.

***Supplementary Figure 3: Box-and-whisker plots comparing fibrotic (#5) vs non fibrotic (#6) specimen of the same patient.*** *The Mann–Whitney U test revealed a statistically significant difference (p = 0.0005, two-tailed). Each bar represents the mean ± SEM. Statistical significance was indicated as follows: *** p = 0.0005 .*

The summary of the descriptive statistics are presented in the table bellow.

***Supplementary Table 2: Descriptive statistics of the Normalized Nanomechanical Measurements in Human Lung Specimens***

|  | **#1** | **#2** | **#3** | **#4** | **#5** | **#6** |
| --- | --- | --- | --- | --- | --- | --- |
| **Minimum** | -1,633 | -1,415 | -0,4224 | -0,9333 | -1,375 | -0,4745 |
| **Median** | 0,0002000 | 0,0001000 | 0,000 | 0,000 | -0,0002000 | 0,000 |
| **Maximum** | 1,342 | 0,8937 | 0,7241 | 1,225 | 0,6872 | 0,8899 |
| **Range** | 2,975 | 2,309 | 1,147 | 2,158 | 2,062 | 1,364 |
| **Mean** | -0,2012 | -0,1766 | 0,06066 | 0,06039 | 0,05624 | 0,05345 |
| **Std. Deviation** | 0,5168 | 0,6065 | 0,2477 | 0,5177 | 0,3463 | 0,3262 |
| **Std. Error of Mean** | 0,008612 | 0,01278 | 0,004300 | 0,008697 | 0,006308 | 0,005570 |
| **Coefficient of variation** | 256,8% | 343,3% | 408,4% | 857,2% | 615,8% | 610,2% |

## **S2.3 Real-time PCR experiments on human specimens**

*COL1A1* mRNA expression was assessed by real time PCR in human samples from PF patients. As shown in **Supplementary Figure 4A** *COL1A1* mRNA expression was significantly elevated in all fibrotic samples compared to the control, indicating active production of collagen I in these patients that could account for the fibrotic status. Also, *COL3A1* mRNA was assessed, but it was found increased in two out of 4tissue specimens, highlighting the heterogeneity among human specimens (Supplementary Figure 4B).


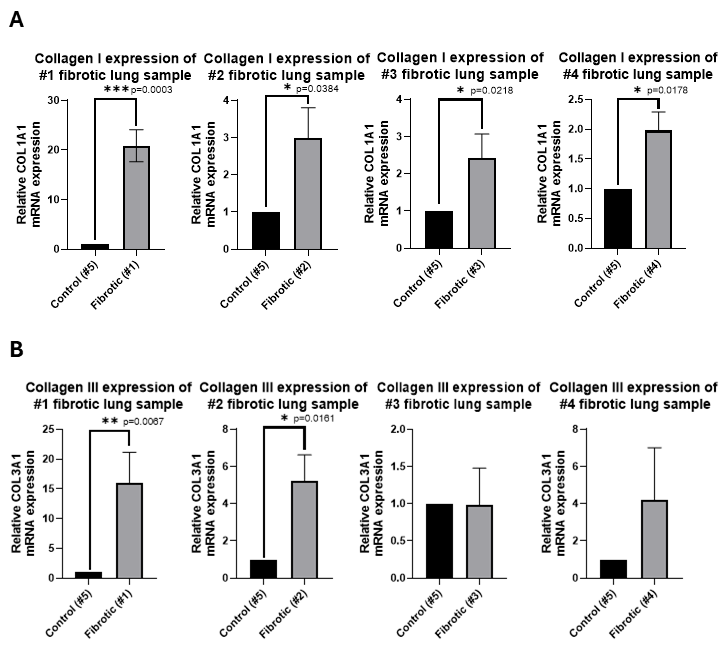


**Supplementary Figure 4: Relative mRNA expression in human specimens with PF. (A)** Relative mRNA expression of COL1A1 (collagen type I). **(B)** Relative mRNA expression of COL3A1 (collagen type III). Each bar represents the mean ± SEM. Asterisks denote statistically significant differences between compared groups (*p < 0.05, **p < 0.01).

***S3. In vitro experiments***

## ***S3.1 In vitro materials and methods***

*S.3.1.1 Cell culturing*

Normal human lung fibroblasts (NHLF, ATCC) Normal human lung fibroblasts (NHLF) (CC-2512, Lonza Bioscience) were cultured in Fibroblast Basal Medium (FBM) supplemented with Fibroblast Growth Medium-2 (FGM-2) NHLF cells were cultured in Fibroblast Basal Medium (FBM) (CC-3131, Lonza Bioscience) supplemented with the Fibroblast Growth Medium-2 (FGM-2) BulletKit (CC-4126, Lonza Bioscience). Cells were maintained in a humidified incubator at 37°C under 5% CO2 and 95% air. Commercially available lung human fibroblasts (FBs), specifically LL 24 (normal FBs) LL24 normal human lung fibroblasts (ATCC, Cat. No. CCL-151), were cultured in F-12K Medium McCoy’s 5A medium, supplemented with 15% FBS and 1% antibiotic/antimycotic in an incubator set to 37°C with 5% CO2.

*S.3.1.2 Cell culturing on* collagen-coated hydrogels

Commercially available collagen-coated hydro-gels with pre-defined stiffness were utilized, as it was presented previously [31]. Specifically, we used collagen-coated hydrogels with stiffness values of 0.5, 1.0 and 4.0 kPa (Petrisoft™ 35 mm Dish Collagen, Cell Guidance Systems). These hydrogels are polyacrylamide-based gels crosslinked with bisacrylamide and coated with type I collagen from bovine skin, bound to 35 mm polystyrene dishes.

*S.3.1.3 AFM experiments on cells*

The AFM experiments were carried out using a commercial AFM (5500 Keysight Technologies, Santa Rosa, CA, USA) equipped with V-shaped soft silicon nitride probes (MLCT-Bio, cantilever C for cells). Given the high-water content of the sample, a Poisson's ratio of ν = 0.5 was assumed. The spring constant was calibrated using the thermal noise method, while the sensitivity calibration (measured as nanometers of cantilever deflection per volt signal from the laser detection system) was performed by obtaining force-versus-distance curves on a Petri dish, which provided a pristine, rigid surface [30, 69]. For the AFM nanoindentation technique a set point of 1 nN normal force for the cells. The Young's modulus was assessed by acquiring 8 × 8 points of force curves in an area of 1 × 1 µm. For the cells this area was selected to be near the centre of the cells. The force–distance curves were processed using the AtomicJ software [35], which was also used to identify the contact point for each curve.

*S.3.1.4 Gene expression*

Total RNA was extracted from cells using Qiazol (Invitrogen), purified using RNeasy mini kit (Qiagen), and transcribed to cDNA using Superscript Reverse Transcriptase (Invitrogen). Quantification of gene expression was performed by Real-Time PCR using CFX96 Real-Time PCR (BioRad). β-actin was used as a housekeeping gene for the normalization. Reactions were conducted in triplicates, and at least 2 independent experiments were performed. Quantification of relative mRNA expression was performed using the *ΔΔCt* method.

## ***S3.2 Results from in vitro experiments***

*In vitro* studies on fibroblast elasticity, morphology, and gene expression (Supplementary Figure 5) were performed using the methodology described in section “S3 In vitro experiments” section. The results revealed that fibrotic fibroblasts (LL97A) exhibit greater deformability (lower stiffness) compared to normal fibroblasts (LL24). Additionally, fibroblasts cultured on progressively stiffer collagen substrates became softer, suggesting a mechano-adaptive response. Morphological analysis showed that fibroblasts adopted a more elongated shape on stiffer substrates, a trait associated with enhanced mrigration [58, 60]. Gene expression analysis further demonstrated a significant downregulation of target genes as substrate stiffness increased from 0.5 to 1 kPa, with no additional changes observed at 4 kPa. Gene expression was performed on targeted genes. More specifically, Yes associated protein -1 (YAP1) is known to be activated by mechanical stimuli, and the RhoGTPases RhoGTPases Ras-Related C3 Botulinum Toxin Substrate 1 (RAC1*),* Ras Homolog Family Member A (RHOA), Cell Division Cycle 42 gene (CDC42), RHO associated coiled coil containing protein kinase (ROCK1), are known to be critically involved in altering actin cytoskeleton affecting cell shape, morphology and motility properties.


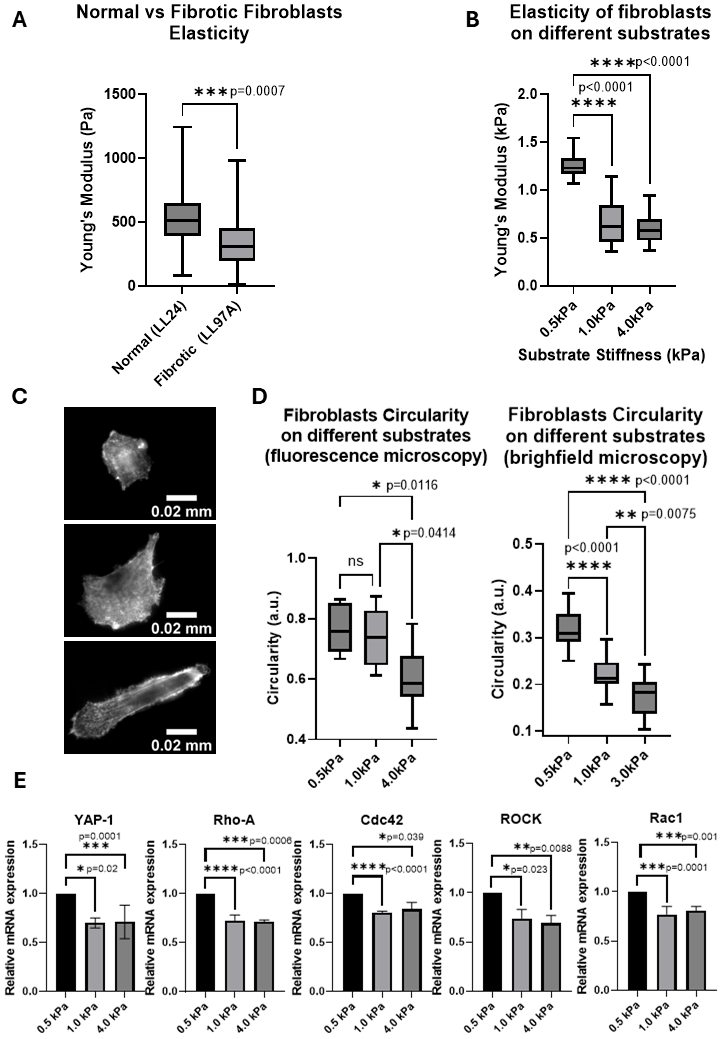


**Supplementary Figure 5**: **In vitro studies on fibroblast elasticity, morphology, and gene expression. A)** Comparison of elasticity (measured as Young's modulus) between normal (LL24, n=38 cells) and fibrotic (LL97A, n=30 cells) fibroblasts, demonstrating that fibrotic fibroblasts exhibit greater deformability (lower stiffness) than normal fibroblasts (p=0.0007). **B)** Young's modulus of fibroblasts cultured on collagen substrates with increasing stiffness, showing that fibroblasts become progressively more deformable (softer) as substrate stiffness increases (n=15). **C)** Representative fluorescence microscopy images of fibroblasts stained with phalloidin to visualize F-actin, acquired using a 100× objective lens. **D)** Quantification of fibroblast circularity from fluorescence microscopy and brighfield images, indicating that fibroblasts adopt a more elongated morphology on stiffer substrates, a characteristic linked to enhanced migratory capabilities (n=20 and n~300 per group, for fluorescence and brighfield microscopy respectively). **E)** Gene expression analysis by real-time PCR, showing a statistically significant downregulation of target genes as substrate stiffness increases from 0.5 to 1 kPa. No further significant changes are observed with an increase in substrate stiffness to 4 kPa. Each bar represents the mean ± SEM. Statistical significance was indicated as follows: *p < 0.05, **p < 0.01, ***p < 0.001, and ****p < 0.0001.

**S4 Ex vivo experiments**

## ***S4.1 Nanomechanical properties of murine lungs during PF progression***

The following figure (Supplementary Figure 6**)** shows the AFM-measured Young’s modulus values at 3, 14, and 21 days following bleomycin administration in both control and treated groups. Specifically, panel A represents data from day 3, panel B from day 14, and panel C from day 21. Data are displayed as individual measurements with mean ± SEM.


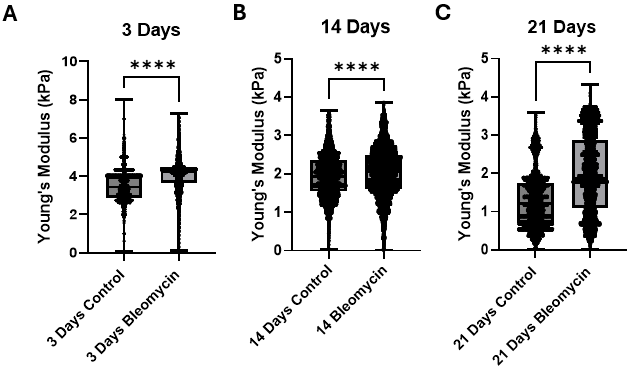


***Supplementary Figure 6****:* ***Young’s modulus values at different time points following bleomycin administration.*** *The graph shows AFM-measured Young’s modulus values at 3, 14, and 21 days post-treatment in control and bleomycin-treated groups.* ***A)*** *Day 3 (4 specimens per group, p < 0.0001),* ***B)*** *Day 14 (7 specimens in the control group, 8 in the bleomycin group, p < 0.0001),* ***C)*** *Day 21 (4 specimens per group, p < 0.0001). Data are presented as individual values with mean ± SEM. Outliers were identified and removed using the ROUT method (Q = 1%). Statistical significance was indicated as follows: *p < 0.05, **p < 0.01, ***p < 0.001, and ****p < 0.0001.*

**S4.2 Correlation Analysis**

S4.2.1 Method

To assess the relationship between tissue stiffness and collagen content during progression of fibrosis, a Spearman rank correlation analysis was performed between Young’s modulus values (obtained with AFM) and collagen content (quantified from picrosirius red-stained lung sections). The analysis was conducted separately for each experimental time point (i.e., 3, 14, and 21 days post-bleomycin treatment). For each time point, data from individual specimens (n = 6–8 per group) were compiled as matched pairs. GraphPad Prism software (version 10.2.0) was used to calculate the Spearman correlation coefficient (r), with two-tailed p-values and a confidence interval of 95%. In this context, a Spearman correlation coefficient (r) greater than 0.8 is considered indicative of a strong positive correlation.

S4.2.2 Results

The correlation between collagen content and tissue stiffness varied across the stages of progression of fibrosis. On day 3, that corresponds to the early inflammatory phase, no correlation was observed (r = –0.095, p = 0.8401), suggesting that mechanical changes had not yet manifested. By day 14, during the active fibrotic stage, a moderate positive correlation emerged (r = 0.486), though it did not reach statistical significance (p = 0.3556), possibly due to limited sample size. On day 21, in the ECM deposition phase, a strong and statistically significant correlation was detected (r = 0.886, p = 0.0333). These results (Supplementary Figure 7) indicate that collagen accumulation becomes a key contributor to increased tissue stiffness at later stages of fibrosis, supporting the biological relevance of AFM-based nanomechanical profiling.


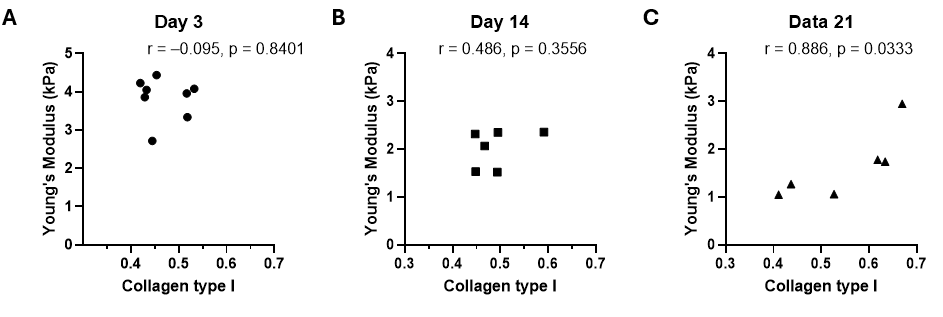


**Supplementary Figure 7: Correlation between collagen content and Young’s modulus at different time points following bleomycin administration**. Spearman correlation plots for (A) Day 3, (B) Day 14, and (C) Day 21. Only the results on Day 21 showed a statistically significant positive correlation (r = 0.886, p = 0.0333).

**S4.3 Cross-Species Normalization and Statistical Analysis of Nanomechanical Signature**

S4.3.1 Method

To enable qualitative comparison of tissue stiffness between fibrotic human and murine lung samples, we performed a z-score normalization on individual AFM-derived Young’s modulus values. Raw values from five human fibrotic specimens and bleomycin-treated murine samples at 14 and 21 days post-treatment were combined into a single dataset. Each individual value was transformed into a z-score using the global mean and standard deviation across all three groups. Z-score normalization allowed the comparison of mechanical trends between species, while accounting for scale differences due to tissue architecture and sample preparation. The normalized data were then analyzed using the Kruskal–Wallis nonparametric test with Dunn’s multiple comparisons post hoc test in GraphPad Prism (version 10.2.0), due to non-normal data distributions confirmed via D'Agostino & Pearson and Kolmogorov–Smirnov normality tests.

S4.3.2 Results

Z-score normalized Young’s modulus values were compared between fibrotic human lung specimens, and murine lungs at 14 and 21 days post-bleomycin treatment (Supplementary Figure 8). The Kruskal–Wallis test revealed a highly significant overall difference across groups (p < 0.0001). Dunn’s post hoc comparisons confirmed significant pairwise differences: both murine groups (Day 14 and Day 21) exhibited significantly higher stiffness than human fibrotic tissues (p < 0.0001), and the Day 14 group showed higher stiffness than the Day 21 group (p < 0.0001). These results highlight measurable mechanical distinctions between species and fibrosis stages, emphasizing the translational value of AFM profiling while acknowledging interspecies variability.

This cross-species comparison of nanomechanical signatures revealed that bleomycin-treated murine lungs exhibit significantly higher stiffness than human fibrotic lungs. The observed interspecies differences likely reflect variations in lung architecture, collagen crosslinking dynamics, and inflammatory response profiles. Importantly, these differences do not undermine the translational relevance of our findings; rather, they emphasize the need to interpret preclinical stiffness data within the context of species-specific biology. The clear distinction between the Day 14 and Day 21 murine groups also reinforces the sensitivity of AFM to detect progressive mechanical remodeling during fibrosis.

While both bleomycin-treated murine groups showed significantly elevated stiffness compared to human fibrotic tissue, the Day 21 group exhibited a smaller rank difference, suggesting that the mechanical profile 21 days post-treatment might better approximate the nanomechanical properties of human pulmonary fibrosis. This highlights that late-stage models could potentially be more representative for translational mechanobiology studies.


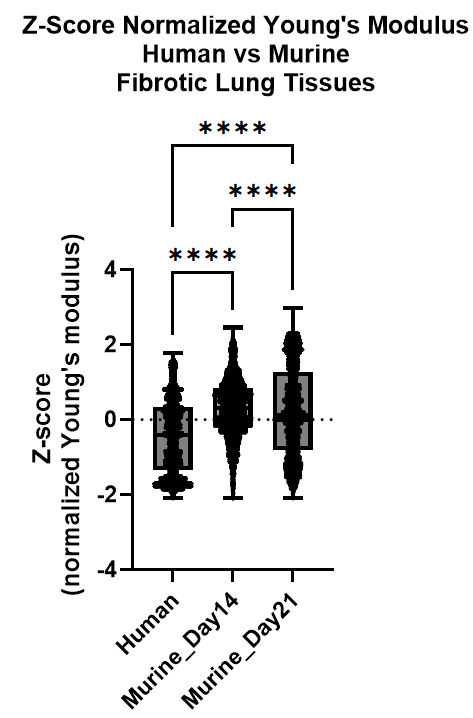


***Supplementary Figure 8****:* ***Comparison of Z-score Normalized Young’s Modulus in Fibrotic Lung Tissue from Humans and Mice.*** *Box-and-whisker plots showing z-score normalized AFM-derived Young’s modulus values from five fibrotic human lung specimens and murine lung samples collected at 14 and 21 days after bleomycin treatment. Each dot represents a single AFM measurement. Statistical analysis using Kruskal–Wallis test followed by Dunn’s multiple comparisons revealed significant differences between all groups: Human vs. Day 14 (****p < 0.0001****), Human vs. Day 21 (****p < 0.0001****), and Day 14 vs. Day 21 (****p < 0.0001****).*

**S4.3 Murine lungs during treatment with bleomycin and Pirfenidone**

To assess the potential of NMFs in monitoring treatment response, we analyzed whether AFM measurements could detect changes in lung tissue mechanics during treatment with pirfenidone, a drug approved for IPF and known to influence tissue remodeling. Lung tissues were collected at 14 and 21 days post-bleomycin, representing key phases of disease progression. The following figure, Supplementary Figure 9, shows representative histological and imaging data from these time points, illustrating collagen remodeling during pirfenidone treatment. Panel A presents picrosirius red-stained images of whole lung sections, reconstructed from multiple high-magnification tiles, while panel B displays SHG microscopy images highlighting collagen distribution and organization.


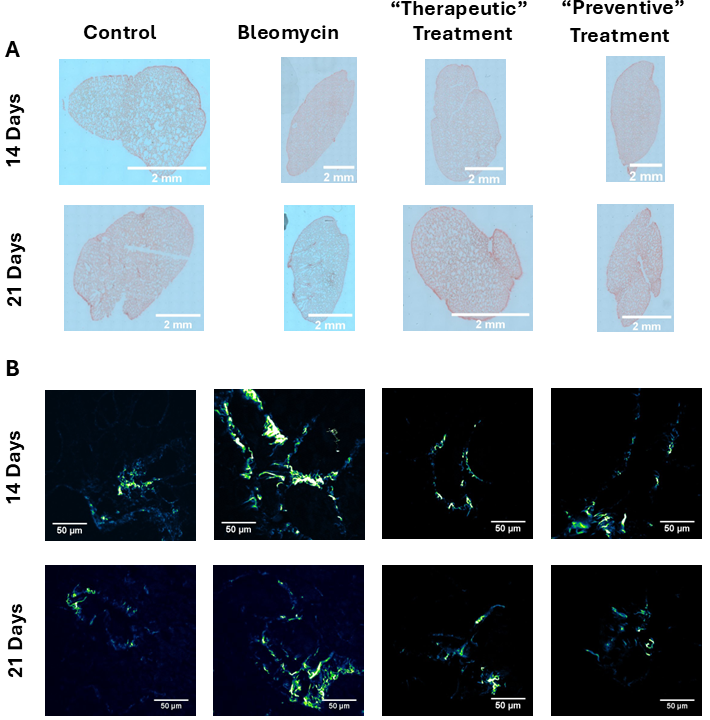


**Supplementary Figure 9: Representative Microscopy Images Following Treatment with Pirfenidone A)** Representative picrosirius red-stained images, each consisting of a different number of tilts from a 20x objective to cover the entire specimen area. (Figures are not the same size because each consists of a different number of same-sized tiles to cover the entire lung section, and each lung section varies in size). **B)** Representative SHG microscopy images showing collagen organization and distribution (scale bar: 50 μm).

**S4.4** **Validation of Nanomechanical Findings in Male Mice**

To further validate the consistency and robustness of the AFM-derived NMFs, we performed additional experiments using a small cohort of male mice. A total of nine male mice were included: three per group (untreated controls, bleomycin-treated, and treated with pirfenidone following bleomycin administration during the “therapeutic” phase). The lungs were collected at the time point of 14 Days.

Young’s modulus values were measured using AFM under identical conditions to those used for the female mouse experiments. The bleomycin-treated male lungs exhibited a marked increase in stiffness and altered nanomechanical spectra compared to untreated controls, consistent with fibrotic remodeling (Supplementary Figure 10 A–C). Treatment with pirfenidone led to a partial normalization of the mechanical profile, reflected in both the distribution of elasticity peaks and the average Young’s modulus values (Supplementary Figure 10 C–D).

To correlate mechanical changes with extracellular matrix remodeling, we performed picrosirius red staining and quantified collagen content using ImageJ. Collagen levels increased significantly in bleomycin-treated lungs compared to controls and were partially reduced following pirfenidone treatment (Supplementary Figure 10 E–F), aligning with the mechanical trends observed in AFM analysis.

To explore the relationship between tissue stiffness and collagen deposition in male mice, a Spearman rank correlation analysis was performed using paired data from AFM-measured Young’s modulus values and collagen content quantified from picrosirius red-stained lung tissue sections. The analysis was conducted in GraphPad Prism (v10.2.0) using a non-parametric, two-tailed Spearman test with a confidence level of 95%. The results in male mice, showed a a statistically significant correlation between collagen content and tissue stiffness (Spearman r = 0.7857, p = 0.0279), indicating that increased collagen deposition is closely associated with higher nanomechanical stiffness. This result supports the consistency of our findings across sexes and further validates the biological relevance of AFM-derived mechanical measurements.

Our findings show that the nanomechanical alterations observed in male mice are in agreement with those observed in female mice. Despite the small sample size, the consistency of the observed patterns—namely, stiffness increases following fibrosis induction and partial reversal with treatment—supports the reproducibility of AFM-based NMFs as potential readouts for disease progression and treatment response. Finally, the fact that we observe similar responses in both sexes further indicates that this is a true response that is sex independent.


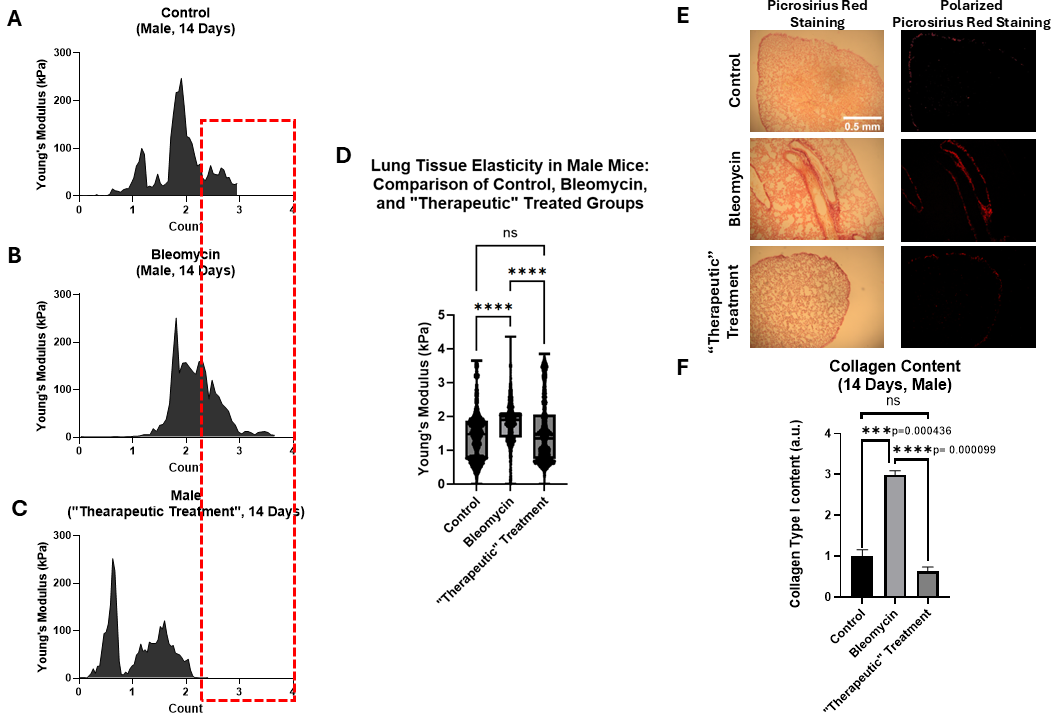


***Supplementary Figure 10: NMFs and Collagen Quantification in Lungs Collected 14 Days Post-Bleomycin Administration During the “Therapeutic Treatment” Phase in Male Mice.*** *A–C) Representative NMFs from AFM elasticity spectra of control, bleomycin-treated male lung tissues, respectively. D) Elasticity spectra illustrating alterations in stiffness distribution, demonstrating the impact of Pirfenidone treatment on lung tissue mechanics. The red square highlights increased elasticity values in the bleomycin (fibrotic) group. D) Quantification of Young’s modulus values across the three groups (control, bleomycin, pirfenidone-treated, n=3 per group), showing increased stiffness after bleomycin administration and partial restoration following Pirfenidone treatment. E) Representative brightfield and polarized light images of picrosirius red-stained lung sections from male mice (scale bar: 0.5 mm). F) Quantification of collagen content from picrosirius red-stained sections reveals elevated collagen levels in the bleomycin group and a significant decrease following Pirfenidone treatment. Data are presented as mean ± SEM. Outliers were removed using the ROUT method (Q = 1%). Statistical comparisons were performed using one-way ANOVA with Bonferroni correction. Statistical significance was indicated as follows: *p < 0.05, **p < 0.01, ***p < 0.001, and ****p < 0.0001*.

1. Melo E, et al. Inhomogeneity of local stiffness in the extracellular matrix scaffold of fibrotic mouse lungs. Journal of the Mechanical Behavior of Biomedical Materials 2014;37:186-95; Júnior C, et al. Multi-Step Extracellular Matrix Remodelling and Stiffening in the Development of Idiopathic Pulmonary Fibrosis. Int J Mol Sci 2023;24. [↑](#footnote-ref-2)
